# Effects of ACSM guideline–based exercise on patients with lung cancer: a systematic review and meta-analysis

**DOI:** 10.3389/fphys.2026.1797432

**Published:** 2026-04-15

**Authors:** Jiangxin Guo, Baofa Wu, Yu Zhang, Jinhan Li, Song Shang, Xu Xu, Dan Xu, Yuxin Nie, Zhu Li, Wuzhuang Sun

**Affiliations:** Department of Respiratory and Critical Care Medicine, The First Hospital of Hebei Medical University, Shijiazhuang, China

**Keywords:** ACSM exercise recommendations, exercise intervention, lung cancer, meta-analysis, quality of life, systematic review

## Abstract

**Objective:**

This study assesses the impact of varying adherence to American College of Sports Medicine (ACSM) guidelines on quality of life and symptom burden in lung cancer patients.

**Methods:**

A systematic search was performed across PubMed, Embase, Web of Science, and Cochrane Library to identify RCTs of exercise in lung cancer. Interventions were classified by adherence to ACSM guidelines for subsequent subgroup analyses. Meta-analyses used random-effects models to pool standardized mean differences (SMDs) and 95% confidence intervals (CIs). Study quality was assessed using the revised Cochrane Risk of Bias tool for randomized trials (RoB 2), and publication bias was evaluated using Begg’s and Egger’s tests.

**Results:**

A total of 32 studies were analyzed, of which 18 demonstrated high adherence to the ACSM guidelines. Meta-analysis demonstrated that exercise interventions significantly improved quality of life (SMD = 0.44, 95% CI: 0.18, 0.70, *p* < 0.001), with a greater effect in the high-adherence group compared with the low or uncertain adherence group (SMD = 0.75 vs. 0.20, *p* = 0.03). Exercise also significantly alleviated fatigue (SMD = −0.50, 95% CI: −0.81, −0.20, *p* = 0.001), with more pronounced improvements in the high-adherence group (SMD = −0.77 vs. −0.11, *p* = 0.004). In addition, exercise significantly reduced anxiety (SMD = −0.63, 95% CI: −1.00, −0.26, *p* < 0.001) and depression (SMD = −0.67, 95% CI: −0.97, −0.38, *p* < 0.001); however, no significant differences were observed between adherence subgroups. Pain was also relieved (SMD = −0.81, 95% CI: −1.49, −0.12, *p* = 0.02), with a greater effect in the high-adherence group (SMD = −1.46 vs. 0.02, *p* = 0.01). Overall evidence shows no clear sleep improvement (SMD = −0.12, 95% CI: −0.37, 0.12, *p* = 0.32), despite borderline significance in the high-adherence subgroup (*p* = 0.05) and near-significant subgroup differences (*p* = 0.06).

**Conclusions:**

Exercise interventions with high adherence to the ACSM guidelines may be more effective in improving quality of life, fatigue, and pain. However, improvements in anxiety and depression do not appear to be dependent on adherence. No significant improvement was observed in sleep quality. Further large-scale studies are warranted.

**Systematic Review Registration:**

https://www.crd.york.ac.uk/prospero/, identifier CRD420251249211.

## 1 Introduction

Lung cancer is one of the most prevalent malignancies worldwide and remains the leading cause of cancer-related mortality (Canadian Cancer Statistics Advisory Committee, 2023). Pathologically, lung cancer is broadly classified into two major subtypes: non-small cell lung cancer (NSCLC) and small cell lung cancer (SCLC). NSCLC is the predominant subtype, accounting for approximately 70%–85% of all cases, whereas SCLC represents about 20%–25%. Epidemiological data indicate that more than 70% of patients are diagnosed at an advanced or metastatic stage, thereby losing the opportunity for curative surgical treatment and resulting in an overall 5-year survival rate of only approximately 15% (Siegel et al., 2011; Jakobsen et al., 2016). The main treatment modalities for lung cancer include surgical resection, chemotherapy, radiotherapy, targeted therapy, and immunotherapy (Gadgeel et al., 2012). Both the disease itself and its treatments give rise to a wide range of symptoms, collectively leading to a marked decline in health-related quality of life among patients with lung cancer. Common symptoms include fatigue, insomnia, anorexia, pain, cough, anxiety, and depression (Iyer et al., 2014). One study reported that more than 90% of patients with advanced NSCLC experience fatigue, loss of appetite, dyspnea, and pain (Iyer et al., 2013). Furthermore, evidence suggests that six months after lobectomy and pneumonectomy, forced expiratory volume in one second (FEV1), an indicator of pulmonary function, declines by 11% and 36%, respectively, while peak oxygen uptake (VO_2_ peak), reflecting overall exercise capacity, decreases by 13% and 28%, representing reductions of up to 40% compared with age-matched healthy individuals (Nezu et al., 1998).

In light of the aforementioned challenges, rehabilitation interventions targeting functional status and quality of life have become increasingly important for patients with lung cancer, in addition to active anticancer treatments. Exercise training, as a nonpharmacological intervention, has gained growing recognition for its role in lung cancer rehabilitation (Granger, 2016). A substantial body of evidence indicates that physical exercise can effectively improve multiple physiological parameters and psychological outcomes in this population. For example, regular exercise has been shown to enhance quality of life by reducing fatigue, improving physical function, and promoting overall well-being (Kangas et al., 2008; Rueda et al., 2011; Wagland et al., 2012; Yates et al., 2013). Moreover, aerobic exercise has been identified as one of the most effective modalities for alleviating anxiety symptoms (Lin and Gao, 2023), while resistance training and mind-body exercises have also been demonstrated to significantly reduce depression and anxiety among lung cancer survivors (Lei et al., 2022).

However, the effects of exercise interventions in patients with lung cancer remain controversial. For instance (Hoffman et al., 2014), reported that exercise improved fatigue, functional status, and quality of life in patients with lung cancer. Similarly (Molassiotis et al., 2021), demonstrated that a 6-week Qigong program alleviated fatigue, dyspnea, and anxiety in this population. In contrast (Dhillon et al., 2017), found that a 2-month physical activity intervention did not result in significant improvements in fatigue or quality of life among patients with advanced lung cancer. Likewise (Temel et al., 2009), observed that a structured exercise program failed to improve depression or anxiety in patients with advanced NSCLC. In addition (Cheville et al., 2013), reported no statistically significant changes in pain or quality of life following an 8-week exercise program in patients with lung cancer. These inconsistencies may be attributable to substantial heterogeneity in exercise intervention protocols (e.g., type, intensity, and duration), patient characteristics (e.g., disease stage and treatment phase), and relatively small sample sizes. Consequently, current research efforts are increasingly focused on clarifying the effectiveness of exercise interventions and identifying optimal exercise prescriptions for this population, including specific modalities, intensities, frequencies, and durations, to achieve truly individualized rehabilitation strategies. Specifically, a methodological distinction must be drawn between conventional, highly heterogeneous exercise programs and interventions based on standardized clinical guidelines. While recent evidence confirms the overall benefits of physical training for lung cancer patients, prior interventions have often lacked standardized dosing parameters. As highlighted by Jiao et al. (2024), pooling diverse exercise regimens obscures the unique therapeutic effects of distinct training modalities, thereby impeding the formulation of tailored rehabilitation protocols in clinical practice. In contrast, exercise protocols structured around the ACSM guidelines provide a quantifiable FITT (Frequency, Intensity, Time, and Type) framework, allowing for a more rigorous assessment of the relationship between exercise dose and clinical benefits (Garber et al., 2011).

The ACSM has developed exercise prescription guidelines for generally healthy adults, providing integrated recommendations on appropriate doses of aerobic, resistance, and flexibility training (Garber et al., 2011). However, in patients with lung cancer, it remains unclear whether exercise interventions that adhere to the ACSM guidelines confer greater benefits in improving quality of life and related symptoms than interventions with low or unspecified adherence. Therefore, this systematic review aims to evaluate and compare the effects of exercise interventions with high adherence versus low or uncertain adherence to the ACSM guidelines on quality of life, fatigue, anxiety, depression, pain, and sleep quality outcomes in patients with lung cancer.

## 2 Materials and methods

This systematic review and meta-analysis was conducted in accordance with the Preferred Reporting Items for Systematic Reviews and Meta-Analyses (PRISMA) guidelines (Page et al., 2021) and the study protocol was prospectively registered with PROSPERO (Registration No.CRD420251249211).

### 2.1 Search strategy

This study was conducted in accordance with the Preferred Reporting Items for Systematic Reviews and Meta-Analyses (PRISMA) guidelines. A comprehensive literature search was performed in the PubMed, Embase, Web of Science, and Cochrane Library databases, covering the period from database inception to October 2025. The search strategy was developed based on the PICOS framework (Population, Intervention, Comparison, Outcomes, and Study design). The primary search included combinations of the following Medical Subject Headings (MeSH) terms and keywords: (“Lung Neoplasms” OR “Pulmonary Neoplasms” OR “Lung Cancer” OR “Pulmonary Cancer”) AND (“Exercise Exercises” OR “Physical Exercise” OR “Aerobic Exercise” OR “Isometric Exercises” OR “Acute Exercise” OR “Exercise Trainings” OR “Physical Activity” OR “Circuit Based Exercise” OR “Circuit Training”). Detailed search strategies for each database are provided in **Supplementary Appendix 1**. In addition, reference lists of relevant review articles and included studies were manually screened to identify additional eligible publications.

### 2.2 Eligibility criteria

Eligibility criteria were defined according to the PICOS framework: (1) Participants: patients aged ≥ 18 years diagnosed with lung cancer, regardless of disease stage or histological type; (2) Interventions: any form of exercise intervention, including aerobic exercise, resistance training, flexibility training, or multimodal exercise programs; (3) Comparisons: control groups receiving usual care, no exercise intervention, or non-exercise interventions; (4) Outcomes: at least one of the following outcomes was reported—quality of life, fatigue, depression, anxiety, pain, or sleep quality; and (5) Study design: RCTs.

Studies were excluded if they met any of the following criteria: (1) animal studies; (2) reviews, case reports, or conference abstracts; (3) studies with insufficient data that could not be obtained by contacting the authors; (4) duplicate publications or studies without full-text availability; and (5) non-randomized designs, including case-control studies, cross-sectional studies, and longitudinal observational studies.

### 2.3 Outcome measures

We defined quality of life (QoL) as the primary outcome, assessing it through validated instruments such as the European Organization for Research and Treatment of Cancer Quality of Life Questionnaire Core 30 (EORTC QLQ-C30), the 36-Item Short Form Survey (SF-36), and other similar scales. In addition to QoL, we examined secondary outcomes related to symptom burden. Specifically, fatigue was evaluated using scales such as the Functional Assessment of Chronic Illness Therapy-Fatigue (FACIT-F/FACT-F), the Brief Fatigue Inventory (BFI), or other validated fatigue questionnaires; pain intensity was measured by instruments such as the Visual Analog Scale (VAS), the Numeric Rating Scale (NRS), or equivalent tools; anxiety and depression were assessed via tools such as the Hospital Anxiety and Depression Scale (HADS), the Self-Rating Anxiety Scale (SAS), the Self-Rating Depression Scale (SDS), or other standardized scales; and sleep quality was rated using questionnaires such as the Pittsburgh Sleep Quality Index (PSQI). For quantitative synthesis, we used the post-intervention mean scores and standard deviations.

### 2.4 Data synthesis and analysis

Two investigators (JXG and BFW) independently screened the literature in accordance with the predefined inclusion and exclusion criteria. Any disagreements were resolved through discussion with a third investigator (YZ). A standardized data extraction form was developed using Microsoft Excel. The following information was extracted: first author, year of publication, country, sample size, participant characteristics (age, sex, and tumor stage), details of the exercise intervention (type, frequency, intensity, duration, and adherence), control group characteristics, outcome measures, and corresponding data. For continuous outcomes, means, standard deviations, and sample sizes at baseline and post-intervention were extracted; when multiple follow-up assessments were reported, only data from the final assessment were included. If outcome data were presented only in graphical form, values were extracted using Engauge Digitizer software. For missing or unclear data, attempts were made to contact the corresponding authors for clarification. When the same outcome was assessed using multiple instruments, data were extracted from the instrument with the highest priority according to a predefined hierarchy.

After data extraction, two investigators (JXG and BFW) independently evaluated the FITT exercise dose (including frequency, intensity, time, and type) and the level of adherence of the exercise interventions for patients with lung cancer, in accordance with the relevant guidelines of the ACSM (**Supplementary Table 1**). Each exercise parameter was scored on a scale of 0–2 points: full compliance with ACSM recommendations was assigned 2 points, parameters categorized as “unable to determine” due to insufficient information were assigned 1 point as a neutral weight, and non-compliance was assigned 0 points. Any discrepancies in scoring were resolved by consensus through consultation with a third investigator (YZ). Based on these scores, the proportion of exercise components meeting the ACSM-recommended dose was calculated for each study. Studies with a compliance proportion of ≥ 75% were classified as having “high adherence,” whereas those with < 75% were categorized as having “low or uncertain adherence”. This specific threshold was informed by established methodological precedents in the field (Fang et al., 2024; Luan et al., 2024; Ye et al., 2024), and the appropriateness of this threshold was further verified by sensitivity analyses. (detailed in **Supplementary Table 4**).

### 2.5 Statistical analysis

Meta-analyses were conducted using Review Manager (RevMan) version 5.4.1. For data reported as non-normally distributed variables in the original studies, where outcomes were presented as medians (M) with interquartile ranges (P25, P75), means and standard deviations were estimated according to the method proposed by (McGrath et al., 2020). Effect sizes were expressed as SMDs with corresponding 95% confidence intervals (95% CI). Given the anticipated heterogeneity across studies in terms of exercise modality, intervention duration, intensity, and adherence to the ACSM guidelines, a random-effects model was prespecified and applied for data pooling. Statistical heterogeneity was assessed using the Higgins *I²* statistic, with *I²* < 50% indicating low heterogeneity and *I²* ≥ 50% indicating high heterogeneity; however, the choice of effect model was not determined by the magnitude of heterogeneity. Subgroup analyses were performed according to the level of adherence to the ACSM guidelines, categorizing studies into high-adherence and low or uncertain-adherence groups, to evaluate the impact of adherence on clinical outcomes and to explore potential sources of heterogeneity. Additionally, to further investigate the sources of heterogeneity, additional subgroup analyses were conducted based on intervention duration (≥ 12 weeks vs. < 12 weeks) and exercise modality (combined vs. single exercise). Results were visualized using forest plots. Publication bias was evaluated using funnel plots in combination with statistical tests at a significance level of *p* < 0.05. Sensitivity analyses were conducted using Stata version 17.0 by sequentially excluding individual studies to assess the robustness and stability of the pooled results.

### 2.6 Quality and certainty of evidence assessment

The methodological quality of the included randomized controlled trials was evaluated using the Cochrane Risk of Bias 2 (RoB 2) tool (Sterne et al., 2019). Two independent pairs of investigators (JXG and BFW; YZ and JHL) followed a three-level hierarchical process. First, specific signaling questions were answered within five domains: (1) randomization process; (2) deviations from intended interventions; (3) missing outcome data; (4) measurement of the outcome; and (5) selection of the reported result. Second, domain-level judgments of “low risk,” “some concerns,” or “high risk” were derived for each domain. Third, the overall risk of bias was determined: a trial was rated as “low risk” only if all domains were at low risk, “some concerns” if at least one domain had concerns but none were at high risk, and “high risk” if any single domain was judged to be at high risk.

Additionally, the certainty of evidence for each outcome was appraised using the GRADE framework. Evidence quality was categorized as high, moderate, low, or very low based on five downgrading factors: risk of bias, inconsistency, indirectness, imprecision, and publication bias. Any discrepancies during the assessment process were resolved through consensus or consultation with a third investigator.

## 3 Results

### 3.1 Literature search

**Figure 1:**
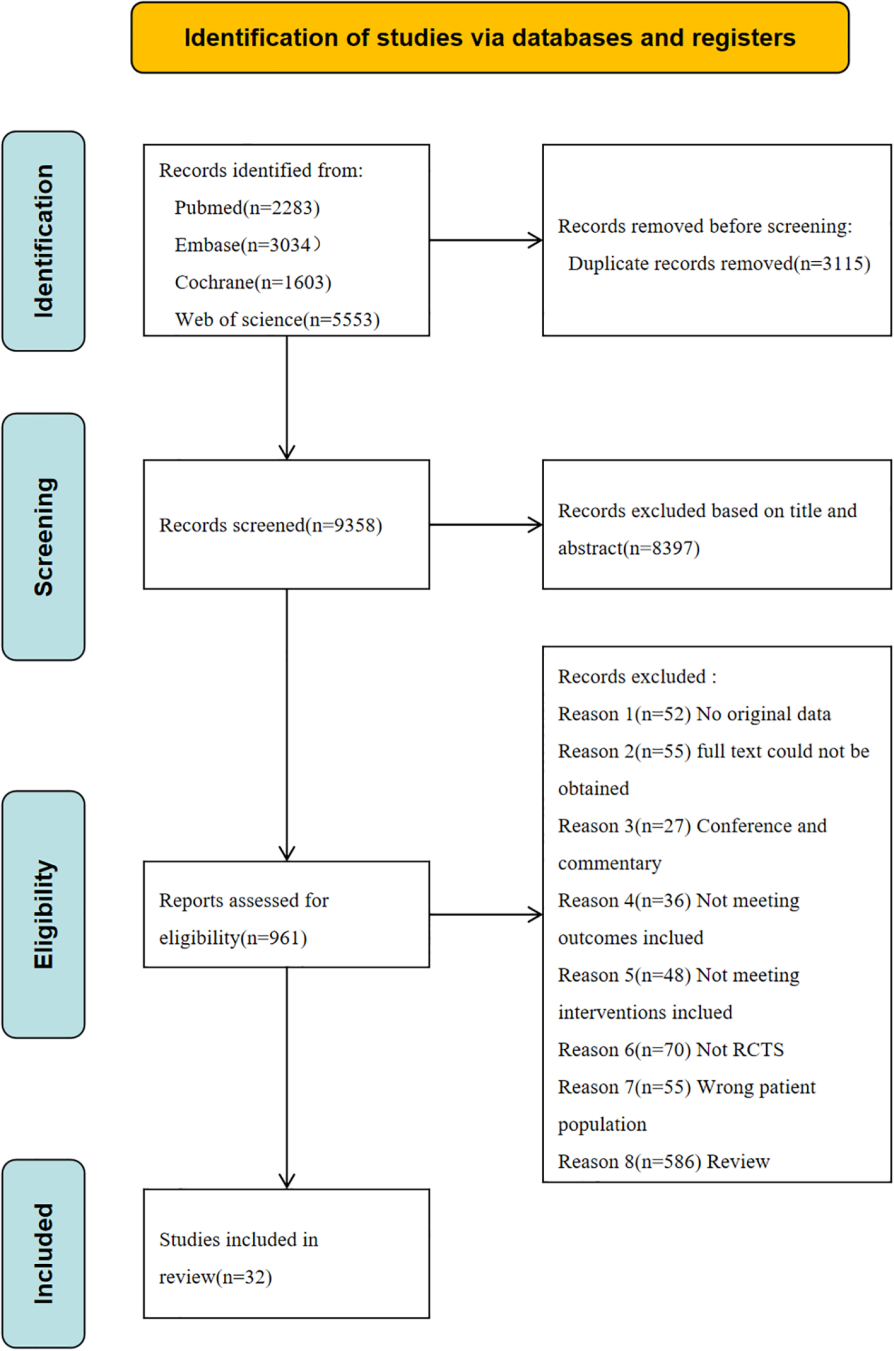
PRISMA study flow diagram.

A systematic search was conducted across four databases: PubMed (n = 2,283), Embase (n = 3,034), the Cochrane Library (n = 1,603), and Web of Science (n = 5,553), yielding a total of 12,473 records. After removal of duplicate publications, 9,358 records remained. Following title and abstract screening, 961 articles were preliminarily identified as potentially eligible. Full-text assessment was subsequently performed, resulting in the exclusion of studies that did not meet the inclusion criteria for the following reasons: review articles (n = 586), non-randomized controlled trials (n = 70), unavailable full texts (n = 55), insufficient original data (n = 52), inappropriate interventions (n = 48), ineligible study populations (n = 55), conference abstracts (n = 27), and failure to report relevant outcomes (n = 36). Ultimately, 32 studies were included in this systematic review (Arbane et al., 2011; Hwang et al., 2012; Henke et al., 2014; Morano et al., 2014; Chen et al., 2015, 2016; Cavalheri et al., 2017; Dhillon et al., 2017; Lai et al., 2017; Egegaard et al., 2019; Jonsson et al., 2019; Messaggi-Sartor et al., 2019; Jiang et al., 2020; Liu et al., 2020; Quist et al., 2020; Sui et al., 2020; Bade et al., 2021; Cheung et al., 2021; Ma et al., 2021; Molassiotis et al., 2021; Ha et al., 2023; Rehman et al., 2023; Xu et al., 2023; Granger et al., 2024; Turan et al., 2024; Wang et al., 2024; Bloch et al., 2025; Lee et al., 2025; Ulrich et al., 2025; Wu et al., 2025; Zhou et al., 2025). The detailed study selection process is presented in **Figure 1**.

### 3.2 Study characteristics

A total of 32 randomized controlled trials were included in this study, comprising 2,820 patients with lung cancer (1,414 in the intervention group and 1,406 in the control group). The mean age of participants ranged from 48.1 to 68.7 years (intervention group: 48.1−68.7 years; control group: 48.3−68.4 years). Except for three studies that did not report sex distribution, the remaining studies indicated that women accounted for 45.98% of participants in the intervention group (54.02% men) and 45.60% in the control group (54.40% men). Regarding geographic distribution, most included studies were conducted in China (n = 14). Other studies were carried out in the United States, Denmark, and Australia (n = 3 each), as well as the United Kingdom, South Korea, Spain, Sweden, Germany, Turkey, Vietnam, Pakistan, and Brazil (n = 1 each).

With respect to study characteristics, the duration of the interventions ranged from 1 week to 12 months. All interventions consisted of supervised and guided exercise programs, encompassing a wide variety of modalities, including cardiorespiratory training, resistance training, flexibility exercises, Tai Chi, and Baduanjin. Specifically, 29 studies incorporated cardiorespiratory training, 15 included resistance training, and 14 involved flexibility training. Control groups primarily received usual care, health education, or non-exercise–based social interaction activities. Outcome assessments focused on patient-reported outcomes, including quality of life (19 studies), fatigue (14 studies), anxiety (14 studies), depression (13 studies), sleep quality (12 studies), and pain (9 studies). Detailed characteristics of the included studies are presented in **Supplementary Table 2**.

### 3.3 Risk of bias

**Figure 2:**
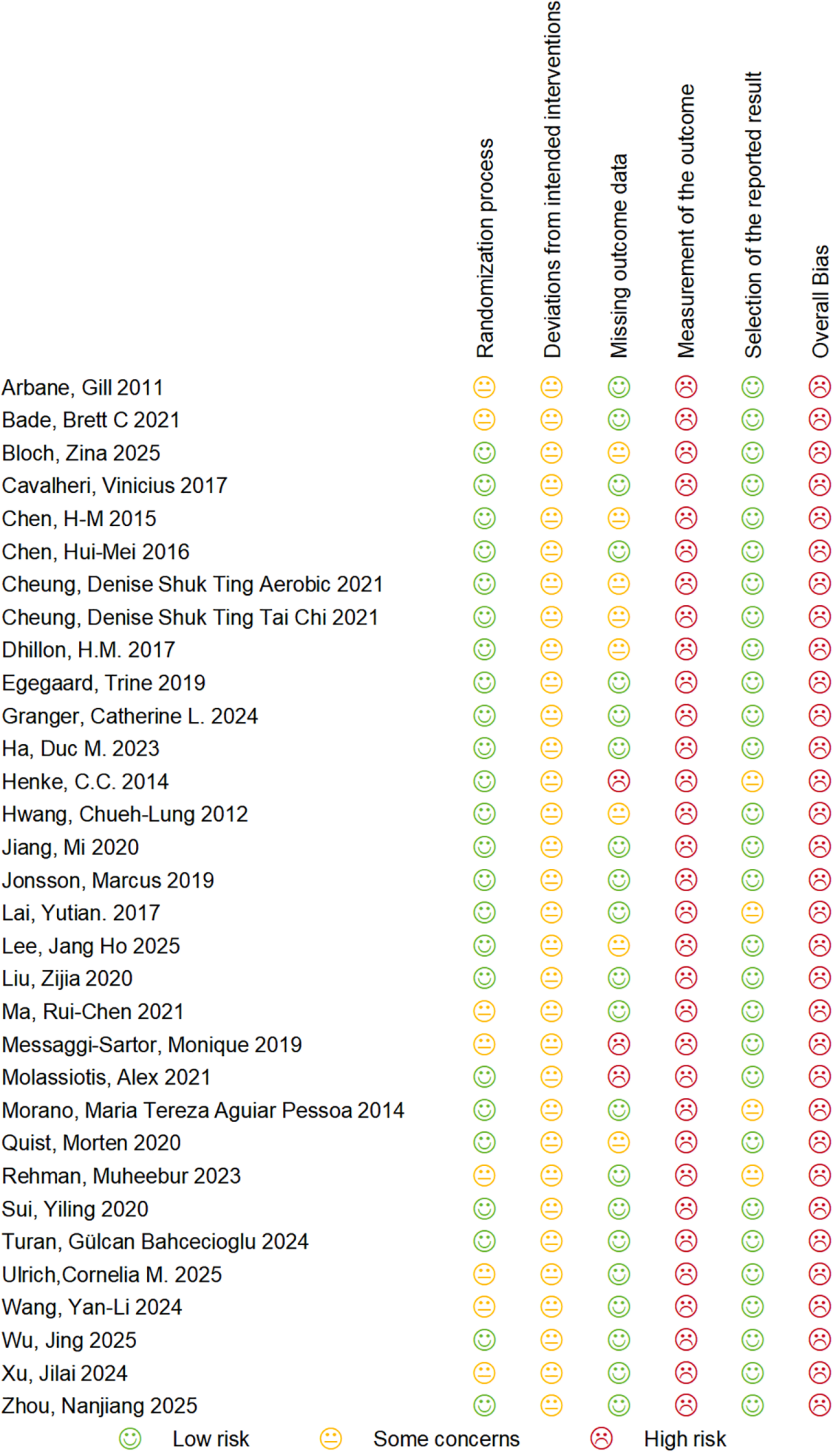
Risk of bias summary.

**Figure 3:**
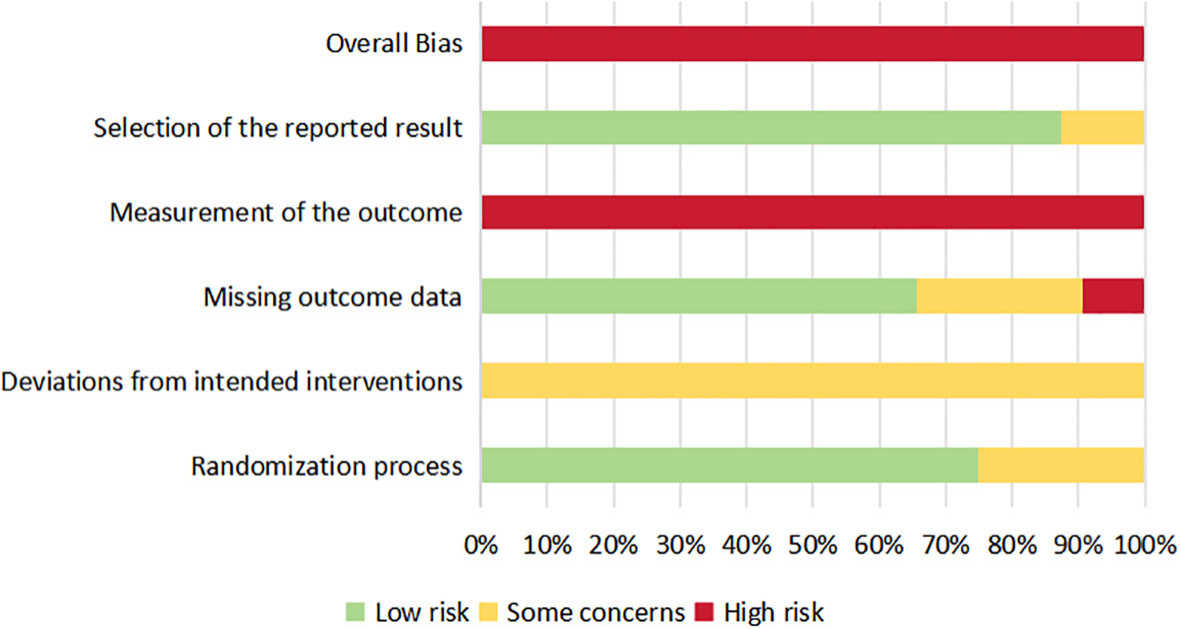
Risk of bias graph.

Assessment with the Cochrane RoB 2 tool showed that all 32 included trials were at an overall high risk of bias (**Figures 2**, **3**). Regarding randomization, approximately 75% of studies were at low risk, while the remaining 25% had some concerns due to unclear concealment procedures. For Domain 2 (deviations from intended interventions), all 32 trials (100%) were rated as having “some concerns” because the nature of exercise interventions precludes blinding of participants and personnel. Domain 3 (missing outcome data) was mostly low risk, though roughly 10% of studies reached high risk due to high attrition. Importantly, every trial (100%) was judged to be at high risk for outcome measurement (Domain 4). This is because the primary endpoints were self-reported via subjective scales without participant blinding, making measurement bias inevitable. Selective reporting (Domain 5) was generally low risk, affecting only about 10% of the trials. Ultimately, the high risk in outcome measurement, combined with the structural inability to blind participants, dictated the overall high risk rating for the entire evidence base. Domain-level results of the risk of bias assessment are summarized in **Figures 2** and **3**.

### 3.4 Adherence to the ACSM guidelines

Among the 32 included studies, 18 demonstrated an adherence level of ≥ 75% to the ACSM-recommended exercise prescription, whereas 14 exhibited adherence levels of < 75% (**Supplementary Table 3**). Low adherence was primarily attributable to discrepancies between the prescribed exercise dose and the ACSM recommendations, as well as insufficient reporting of exercise prescription details that precluded accurate assessment. When stratified by outcome measures, the distribution of adherence levels was as follows: among studies assessing quality of life, 9 reported high adherence to the ACSM guidelines and 10 reported low or uncertain adherence; among studies evaluating fatigue, 9 demonstrated high adherence and 5 showed low or uncertain adherence; among studies examining depression, 8 reported high adherence and 5 reported low or uncertain adherence; among studies assessing anxiety, 9 demonstrated high adherence and 5 showed low or uncertain adherence; among studies evaluating pain, 5 reported high adherence and 4 reported low or uncertain adherence; and among studies assessing sleep quality, 8 demonstrated high adherence and 4 showed low or uncertain adherence.

### 3.5 Meta-analysis

#### 3.5.1 Quality of life

**Figure 4:**
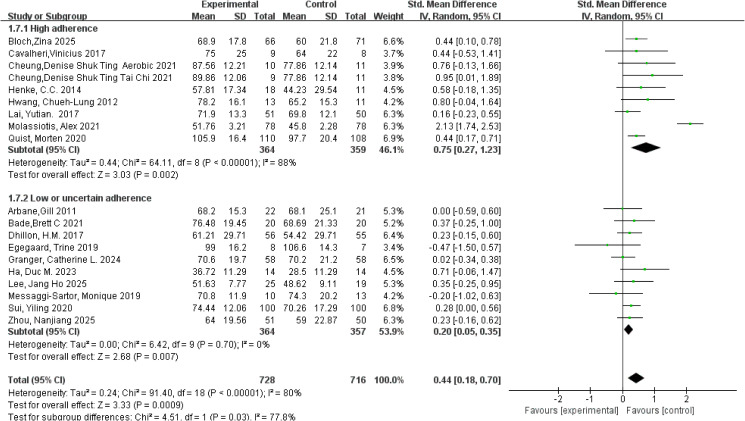
Forest plot of meta-analysis on the effect of exercise on Quality of Life in Lung Cancer patients.

**Figure 5:**
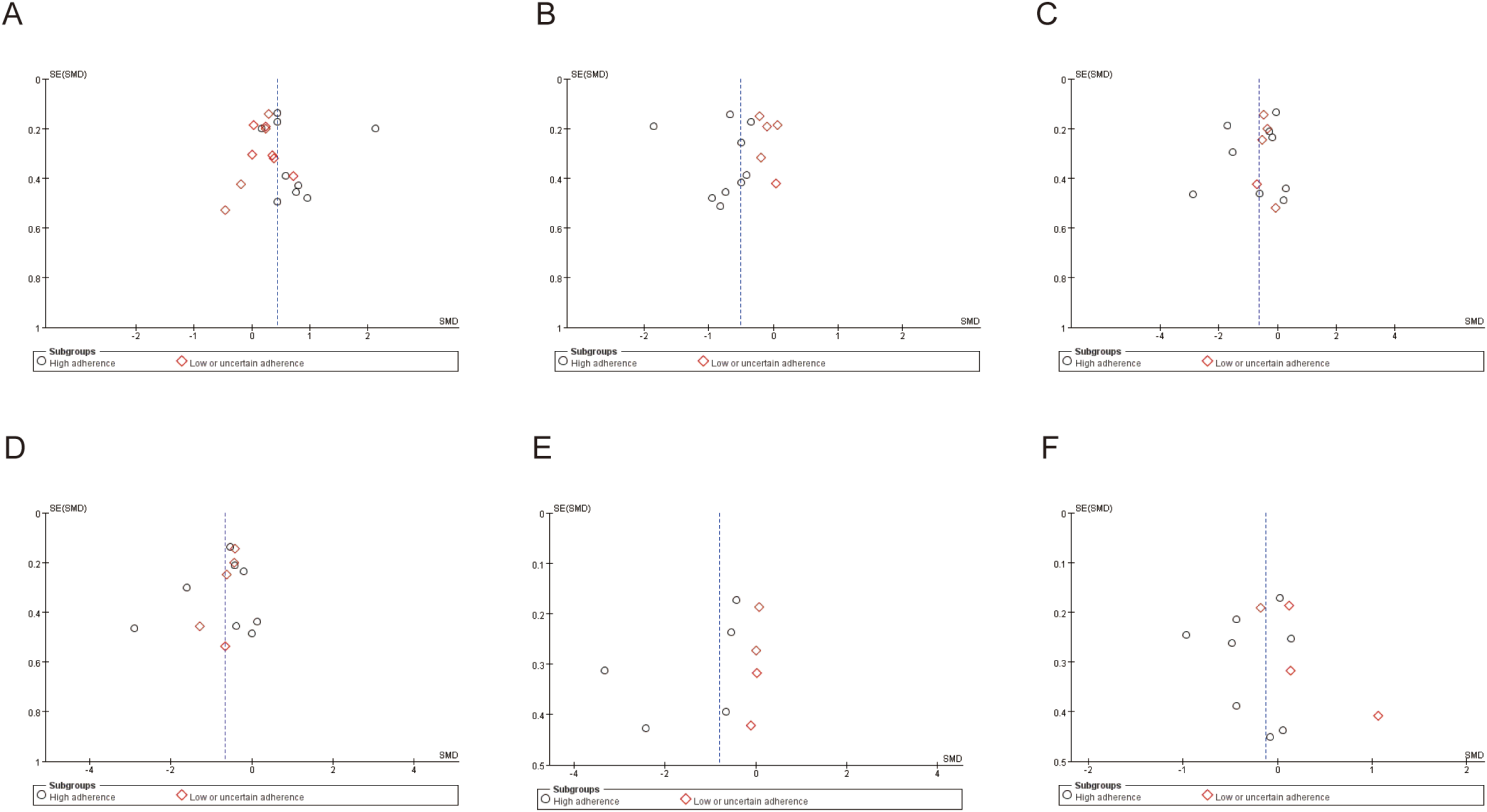
Funnel plots of the meta-analysis on the effect of exercise in lung cancer patients. **(A)** Quality of life; **(B)** Fatigue; **(C)** Anxiety; **(D)** Depression; **(E)** Pain; **(F)** Sleep quality.

Quality of life was primarily assessed using the EORTC QLQ-C30, the SF-36, and the Functional Assessment of Cancer Therapy-Lung (FACT-L) instruments, with consistent use of these measures over time. Nineteen studies involving a total of 1,444 participants were included in the meta-analysis, and pooled effect sizes were calculated using a random-effects model. Substantial heterogeneity was observed for the quality-of-life outcome (*I²* = 80%, *p* < 0.001). The meta-analysis demonstrated that exercise interventions had a significant positive effect on quality of life in patients with lung cancer, with a pooled standardized mean difference (SMD) of 0.44 (95% CI: 0.18, 0.70, *p* < 0.001). Subgroup analyses based on adherence to the ACSM guidelines revealed that the high-adherence subgroup (≥ 75%) showed a pooled SMD of 0.75 (95% CI: 0.27, 1.23, *p* = 0.002), whereas the low or uncertain-adherence subgroup demonstrated a pooled SMD of 0.20 (95% CI: 0.05, 0.35, *p* = 0.007). The between-subgroup difference was statistically significant, indicating that higher adherence to the ACSM guidelines was associated with greater improvements in quality of life (*p* = 0.03; **Figure 4**). Accordingly, exercise interventions yielded superior quality-of-life benefits in studies with high adherence compared with those with lower adherence. Within-subgroup heterogeneity analyses showed high heterogeneity in the high-adherence subgroup (*I²* = 88%) and low heterogeneity in the low or uncertain-adherence subgroup (*I²* = 0%). Assessment of publication bias indicated that the funnel plot was visually symmetrical (**Figure 5A**), and the results of Begg’s test (*p* = 0.196) and Egger’s test (*p* = 0.934) were consistent, suggesting no significant publication bias.

#### 3.5.2 Fatigue

**Figure 6:**
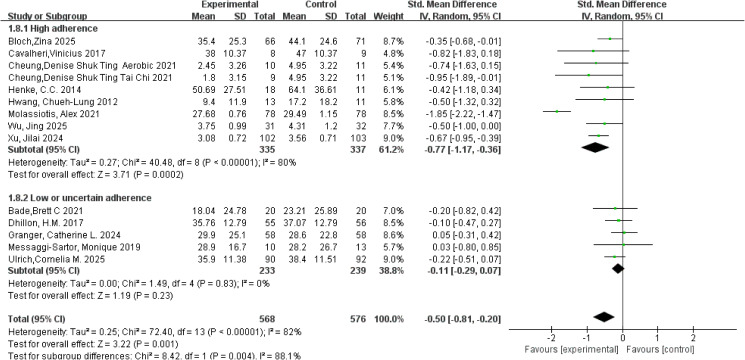
Forest plot of meta-analysis on the effect of exercise on fatigue in lung cancer patients.

Fatigue was primarily assessed using the fatigue subscale of the EORTC QLQ-C30, the FACIT-F/FACT-F, the BFI, and the Revised Piper Fatigue Scale (PFS), with relatively consistent use of these instruments over time. A meta-analysis of 14 studies involving 1,144 participants was conducted using a random-effects model to pool effect sizes. Considerable heterogeneity was observed for the fatigue outcome (*I²* = 82%, *p* < 0.001). Overall, the analysis demonstrated that exercise interventions significantly reduced fatigue levels in patients with lung cancer, with a pooled SMD of −0.50 (95% CI: −0.81, −0.20, *p* = 0.001). Subgroup analyses based on adherence to the ACSM guidelines indicated that studies with high adherence showed a pooled SMD of −0.77 (95% CI: −1.17, −0.36, *p* < 0.001), whereas studies with low or uncertain adherence demonstrated a pooled SMD of −0.11 (95% CI: −0.29, 0.07, *p* = 0.23). The between-subgroup difference was statistically significant, suggesting that higher adherence to the ACSM guidelines was associated with more pronounced fatigue alleviation (*p* = 0.004; **Figure 6**). Within-subgroup heterogeneity analyses revealed high heterogeneity in the high-adherence subgroup (*I²* = 80%) and low heterogeneity in the low or uncertain-adherence subgroup (*I²* = 0%). Assessment of publication bias showed that the funnel plot was visually symmetrical (**Figure 5B**), and the results of Begg’s test (*p* = 0.171) and Egger’s test (*p* = 0.858) were consistent, indicating no evidence of significant publication bias.

#### 3.5.3 Anxiety

**Figure 7:**
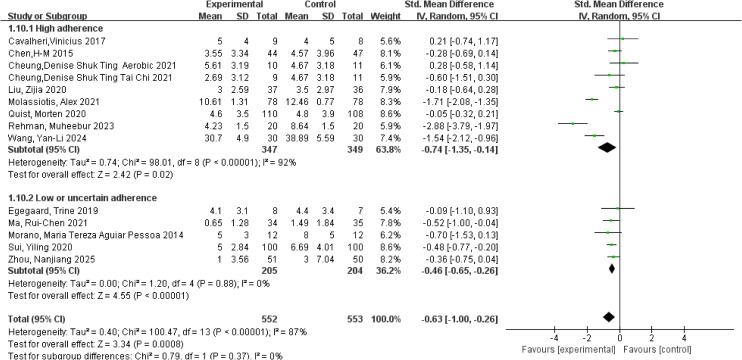
Forest plot of meta-analysis on the effect of exercise on anxiety in lung cancer patients.

Anxiety was primarily assessed using the Hospital Anxiety and Depression Scale-Anxiety (HADS-A), the SAS, and the Generalized Anxiety Disorder-7 (GAD-7), with relatively consistent use of these instruments across studies. A meta-analysis of 14 studies involving 1,105 participants was conducted using a random-effects model. Substantial heterogeneity was observed for the anxiety outcome (*I²* = 87%, *p* < 0.001). The pooled analysis demonstrated that exercise interventions significantly reduced anxiety levels in patients with lung cancer, with an overall SMD of −0.63 (95% CI: −1.00, −0.26, *p* < 0.001). Subgroup analyses based on adherence to the ACSM guidelines showed that studies with high adherence yielded a pooled SMD of −0.74 (95% CI: −1.35, −0.14, *p* = 0.02), whereas studies with low or uncertain adherence demonstrated a pooled SMD of −0.46 (95% CI: −0.65, −0.26, *p* < 0.001). However, the between-subgroup difference did not reach statistical significance, indicating that higher adherence to the ACSM guidelines could not be confirmed to confer additional benefits in anxiety reduction (*p* = 0.37; **Figure 7**). Within-subgroup heterogeneity analyses revealed high heterogeneity in the high-adherence subgroup (*I²* = 92%) and low heterogeneity in the low or uncertain-adherence subgroup (*I²* = 0%). Assessment of publication bias indicated that the funnel plot was visually symmetrical (**Figure 5C**), and the results of Begg’s test (*p* = 0.622) and Egger’s test (*p* = 0.514) were consistent, suggesting no significant publication bias.

#### 3.5.4 Depression

**Figure 8:**
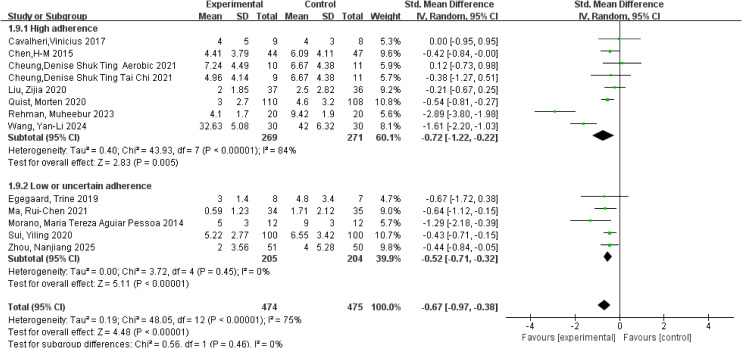
Forest plot of meta-analysis on the effect of exercise on depression in lung cancer patients.

Depression was primarily assessed using the Hospital Anxiety and Depression Scale-Depression (HADS-D), the SDS, and the Patient Health Questionnaire-9 (PHQ-9), with consistent use of these instruments across studies. A meta-analysis of 13 studies involving 949 participants was conducted using a random-effects model to pool effect sizes. Considerable heterogeneity was observed for the depression outcome (*I²* = 75%, *p* < 0.001). The pooled analysis demonstrated that exercise interventions significantly reduced depressive symptoms in patients with lung cancer, with an overall SMD of −0.67 (95% CI: −0.97, −0.38, *p* < 0.001). Subgroup analyses based on adherence to the ACSM guidelines showed that studies with high adherence yielded a pooled SMD of −0.72 (95% CI: −1.22, −0.22, *p* = 0.005), whereas studies with low or uncertain adherence demonstrated a pooled SMD of −0.52 (95% CI: −0.71, −0.32, *p* < 0.001). The between-subgroup difference did not reach statistical significance, indicating that higher adherence to the ACSM guidelines could not be confirmed to provide additional benefits in reducing depressive symptoms (*p* = 0.46; **Figure 8**). Within-subgroup heterogeneity analyses revealed high heterogeneity in the high-adherence subgroup (*I²* = 84%) and low heterogeneity in the low or uncertain-adherence subgroup (*I²* = 0%). Assessment of publication bias showed that the funnel plot was visually symmetrical (**Figure 5D**), and the results of Begg’s test (*p* = 0.393) and Egger’s test (*p* = 0.263) were consistent, suggesting no evidence of significant publication bias.

#### 3.5.5 Pain

**Figure 9:**
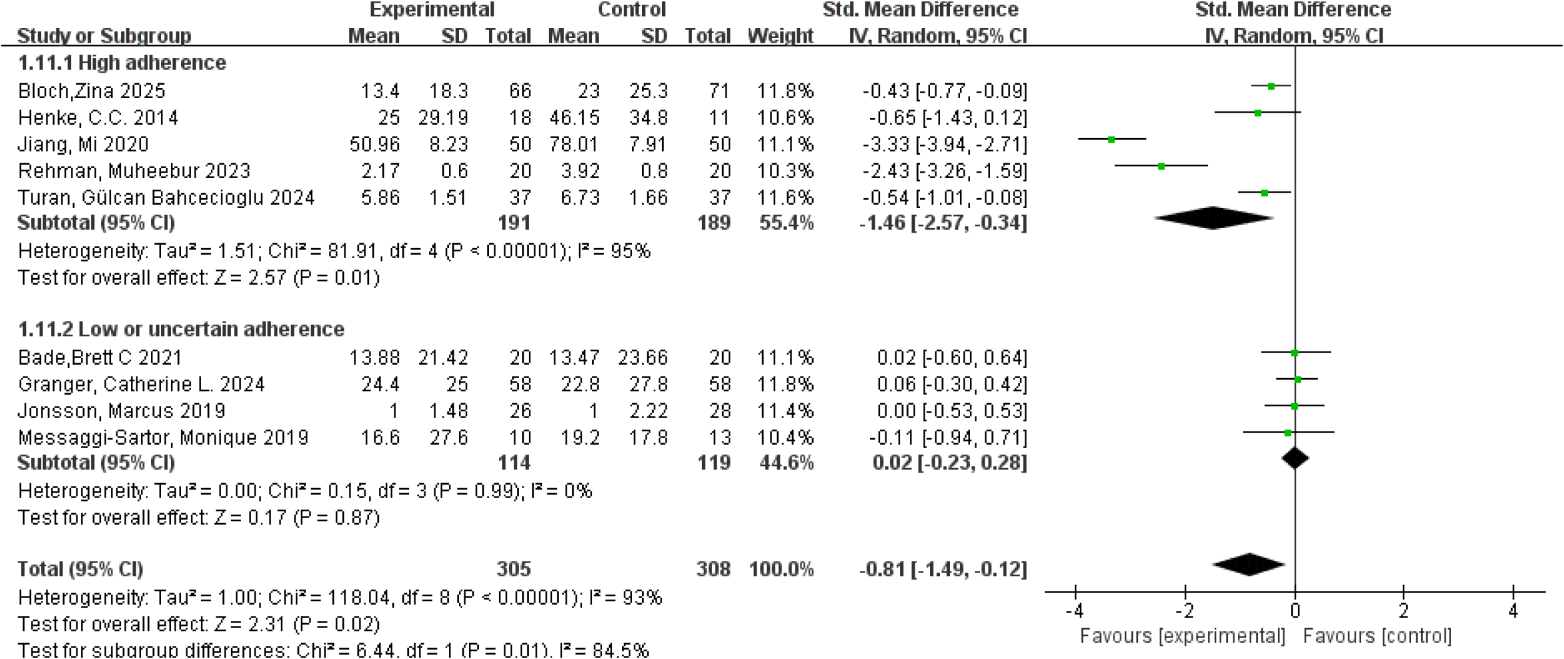
Forest plot of meta-analysis on the effect of exercise on pain in lung cancer patients.

Pain was primarily assessed using the pain subscale of the EORTC QLQ-C30, the VAS, and the NRS, with consistent application of these instruments across studies. A meta-analysis of 9 studies involving 613 participants was conducted using a random-effects model. Substantial heterogeneity was observed for the pain outcome (*I²* = 93%, *p* < 0.001). The pooled analysis indicated that exercise interventions significantly reduced pain intensity in patients with lung cancer, with an overall SMD of −0.81 (95% CI: −1.49, −0.12, *p* = 0.02). Subgroup analyses based on adherence to the ACSM guidelines revealed that studies with high adherence demonstrated a pooled SMD of −1.46 (95% CI: −2.57, −0.34, *p* = 0.01), whereas studies with low or uncertain adherence showed a pooled SMD of 0.02 (95% CI: −0.23, 0.28, *p* = 0.87). The between-subgroup difference was statistically significant, indicating that higher adherence to the ACSM guidelines was associated with more pronounced pain relief. Accordingly, exercise interventions yielded superior pain reduction in studies with high adherence compared with those with lower adherence (*p* = 0.01; **Figure 9**). Within-subgroup heterogeneity analyses showed high heterogeneity in the high-adherence subgroup (*I²* = 95%) and low heterogeneity in the low or uncertain-adherence subgroup (*I²* = 0%). Assessment of publication bias for pain showed a visually symmetrical funnel plot (**Figure 5E**), and both Begg’s test (*p* = 0.211) and Egger’s tests (*p* = 0.270) yielded non-significant results. However, these findings should be interpreted with caution, as the limited number of studies (n = 9) and the presence of high heterogeneity (*I²* = 93%) may reduce the statistical power of these tests to detect potential bias.

#### 3.5.6 Sleep quality

**Figure 10:**
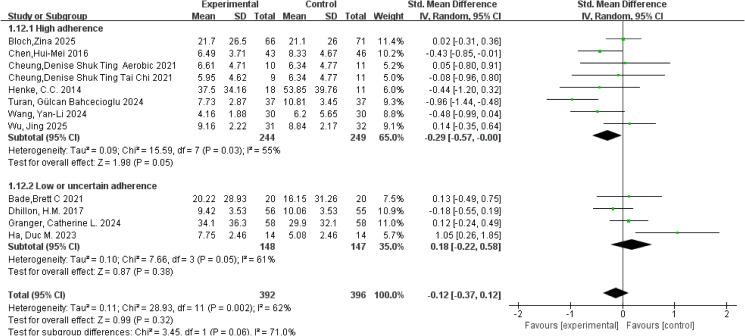
Forest plot of meta-analysis on the effect of exercise on sleep quality in lung cancer patients.

Sleep quality was primarily assessed using the PSQI and the insomnia subscale of the EORTC QLQ-C30, with consistent application of these instruments across studies. A meta-analysis of 12 studies involving 788 participants was conducted using a random-effects model. Moderate heterogeneity was observed for the sleep quality outcome (*I²* = 62%, *p* = 0.002). The pooled analysis showed no clear overall improvement in sleep quality for patients with lung cancer, with an overall SMD of −0.12 (95% CI: −0.37, 0.12, *p* = 0.32). However, subgroup analysis based on ACSM adherence revealed that the high-adherence subgroup showed borderline significance, yielding a pooled SMD of −0.29 (95% CI: −0.57, −0.00, *p* = 0.05), whereas the low or uncertain adherence group showed an SMD of 0.18 (95% CI: −0.22, 0.58, *p* = 0.38). Notably, the between-group difference reached near significance (*p* = 0.06; **Figure 10**), suggesting that a dose-related trend may exist but remains uncertain. Within-subgroup heterogeneity analyses indicated moderate heterogeneity in both the high-adherence and low or uncertain-adherence subgroups (*I²* = 55% and 61%, respectively). Assessment of publication bias revealed that the funnel plot was visually symmetrical (**Figure 5F**), and the results of Begg’s test (*p* = 1.000) and Egger’s test (*p* = 0.714) were consistent, indicating no evidence of significant publication bias.

#### 3.5.7 Sensitivity analysis

**Figure 11:**
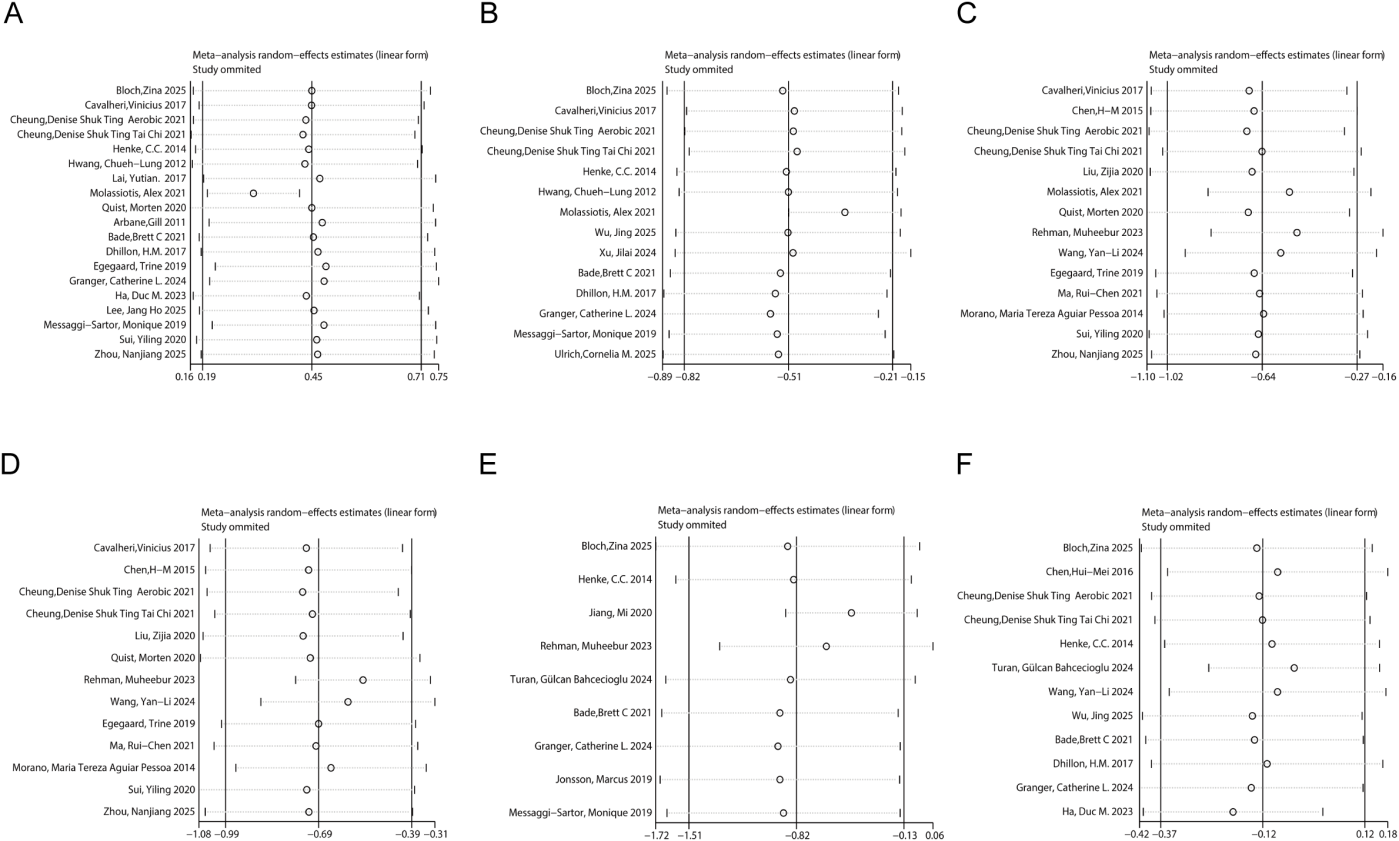
Sensitivity analysis of the effect of exercise on outcomes in lung cancer patients. **(A)** quality of life; **(B)** fatigue; **(C)** anxiety; **(D)** depression; **(E)** pain; **(F)** sleep quality.

To explore potential sources of heterogeneity and verify the stability of the results, two types of sensitivity analyses were performed. First, a leave-one-out analysis indicated that no individual study had a disproportionate influence on the overall pooled estimates, supporting the robustness of the findings (**Figure 11**). Second, the appropriateness of the 75% adherence threshold was evaluated by comparing it with alternative cut-offs (60% and 80%). The results demonstrated that the 75% threshold provided the most consistent discriminatory power, whereas other thresholds led to less conclusive findings or extreme sample size imbalances in certain outcomes (**Supplementary Table 4** and **Supplementary Figures 1–1** to **1**-**12**).

#### 3.5.8 Exploration of heterogeneity

To investigate the sources of the observed statistical heterogeneity, additional subgroup analyses were performed based on intervention duration (≥ 12 weeks vs. < 12 weeks) and exercise modality (combined vs. single exercise), as detailed in **Supplementary Table 5** and **Supplementary Figures 2–1** to **2**-**12**. For Quality of Life, the overall heterogeneity (*I²* = 80%, *p* < 0.001) was markedly reduced within the ≥ 12 weeks subgroup (*I²* = 7%, *p* = 0.37) and eliminated in the combined exercise subgroup (*I²* = 0%, *p* = 0.60). Regarding Fatigue, heterogeneity was resolved within the combined exercise subgroup (*I²* = 0%, *p* = 0.50). Similarly, for psychological outcomes, *I²* for Depression was absent (*I²* = 0%, *p* = 0.70) in the ≥ 12 weeks subgroup, while for Anxiety, it decreased to 41% (*p* = 0.15) in the same category. These findings suggest that intervention duration and exercise modality are key contributors to the variance in clinical efficacy across the included studies.

#### 3.5.9 Certainty of evidence

The certainty of evidence for all outcomes was appraised using the GRADE framework and is summarized in **Supplementary Table 6**. For the primary outcome, quality of life, the certainty was rated as “low” due to serious risk of bias (lack of participant blinding) and serious inconsistency (*I²* = 80%). Similarly, the certainty for fatigue, anxiety, and depression was graded as “low”, primarily downgraded for risk of bias in open-label trials and high statistical heterogeneity (*I²* = 75–87%). Evidence certainty for pain and sleep quality was further downgraded to “very low”. In addition to concerns regarding bias and inconsistency, these outcomes demonstrated serious imprecision, as evidenced by small total sample sizes (below the optimal information size) or 95% confidence intervals that crossed the null value (for sleep quality). Overall, while exercise interventions showed statistically significant benefits for most health outcomes, the certainty of evidence remains low to very low, necessitating a cautious interpretation of these findings.

## 4 Discussion

This meta-analysis systematically evaluated the effects of exercise interventions based on the ACSM guidelines on outcomes in patients with lung cancer, including 32 studies encompassing 2,820 patients from 13 countries. The findings demonstrated that exercise significantly improved quality of life, reduced cancer-related fatigue, alleviated depressive and anxiety symptoms, and effectively relieved pain. A key strength and innovation of this study lies in the first quantitative assessment of adherence to the ACSM guidelines as a standardized criterion for exercise interventions, followed by adherence-based subgroup analyses. Our findings suggest that high-adherence exercise interventions were more effective in improving quality of life, fatigue, and pain than those with low or uncertain adherence. Meanwhile, no clear adherence-dependent benefits were observed for anxiety and depression; however, the results for sleep quality suggest a possible dose-related trend, although the evidence remains inconclusive at this stage. These findings, however, must be interpreted within a cautious framework due to several methodological constraints. Firstly, the theoretical framework for assessing ACSM adherence has inherent limitations, as pooling confirmed low-dose and under-reported interventions may dilute true clinical effects. Secondly, significant heterogeneity in measurement scales across the included trials complicates the direct comparison of outcomes. Finally, given the generally low-to-very-low certainty of evidence (GRADE), our conclusions should be regarded as preliminary rather than definitive. We have prioritized these considerations at the outset to provide a transparent context for the following detailed analysis.

This meta-analysis demonstrates that exercise interventions can improve both physical and psychological outcomes in patients with lung cancer, which is consistent with findings from previous studies (Barrera-Garcimartín et al., 2023; Hu et al., 2025; Zhao et al., 2025). Through adherence-based subgroup analyses, our results further indicate that these beneficial effects are symptom specific, and the underlying mechanisms may be related to differential dose dependence of various symptoms in response to exercise. Specifically, improvements in quality of life may arise from multiple synergistic mechanisms. For outcomes such as quality of life, fatigue, and pain—where significant advantages were observed in the high-adherence groups—the benefits appear to be primarily driven by physiological stress responses induced by exercise doses consistent with ACSM recommendations (Campbell et al., 2019). Exercise directly enhances cardiopulmonary function and muscular strength (Cavalheri and Granger, 2017), thereby improving overall physical capacity, and such structural and functional adaptations are likely to require a minimum threshold of exercise intensity (e.g., moderate to vigorous intensity as recommended by the ACSM) and sufficient cumulative duration (Edvardsen et al., 2015). In terms of fatigue reduction, exercise may exert its effects by modulating systemic inflammatory pathways (e.g., reducing levels of IL-6 and TNF-α) (Meneses-Echávez et al., 2016) and by improving mitochondrial biogenesis and metabolic efficiency in skeletal muscle (Clemente-Suárez et al., 2023); these favorable molecular and cellular adaptations are more likely to be fully activated by regular exercise performed at adequate intensity (Pedersen, 2019). In contrast, the beneficial effects of exercise on depression and anxiety appear to exhibit a lower threshold, potentially mediated by regulation of neuroplasticity and neurotransmitter systems, including enhanced release of dopamine, β-endorphins, and serotonin, which can lead to relatively rapid improvements in mood even at lower exercise doses (Basso and Suzuki, 2017). With respect to pain management, exercise may involve a combination of dose-dependent physiological improvements (such as reductions in nociceptive mediators) and endogenous analgesic mechanisms (such as endorphin release) (Sluka et al., 2018), which together may partly explain why exercise interventions with higher adherence to the ACSM guidelines yielded more pronounced pain relief (Naugle et al., 2014).

Based on the adherence-based subgroup analyses, the mechanisms discussed above provide a plausible explanation for the observed findings. For quality of life, fatigue, and pain, the high-adherence subgroup demonstrated clear advantages, suggesting that the exercise frequency, intensity, and duration recommended by the ACSM guidelines may represent necessary conditions for inducing sufficient physiological stress to trigger dose-dependent beneficial adaptations (Campbell et al., 2019), such as anti-inflammatory responses (Sluka et al., 2018), metabolic improvements, and neuromuscular adaptations. Accordingly, in clinical practice, maximizing improvements in these functional outcomes and somatic symptoms may require moving beyond general physical activity advice toward the prescription and strict implementation of structured exercise programs with clearly defined doses and high adherence (Buffart et al., 2017). In contrast, improvements in depression and anxiety did not exhibit a comparable dependence on adherence (Rebar et al., 2015). One possible explanation is that the neurophysiological mechanisms underlying these psychological outcomes (e.g., neurotransmitter release) and related pathways (e.g., attentional diversion and pleasure induction) have relatively low activation thresholds, such that even regular low-intensity physical activity (e.g., daily walking) can elicit meaningful acute effects (Peluso and Guerra, 2005; Basso and Suzuki, 2017). Moreover, psychological outcomes are influenced by numerous non-exercise-related factors, including social support, economic stress, illness perception, and pharmacological treatments (Richardson et al., 2017; Chan et al., 2019); these confounding influences may statistically obscure subtle differences between exercise doses (Carayol et al., 2013). Consequently, although higher exercise doses may confer additional physiological benefits, their marginal effects on psychological outcomes at the population level may be outweighed by stronger confounding factors, thereby resulting in non-significant between-group differences based on exercise dose in the present study.

The exercise guidelines issued by the ACSM represent authoritative standards designed to enhance cardiorespiratory fitness, muscular strength, and flexibility in healthy adults, and their effectiveness has been well established in the rehabilitation of various chronic conditions, such as Parkinson’s disease and osteoarthritis (Mak et al., 2017; Lawford et al., 2024); however, their role in lung cancer rehabilitation has not been systematically evaluated. This study is the first meta-analysis to address this gap, demonstrating that, compared with exercise interventions with low or uncertain adherence, programs that strictly follow the exercise doses recommended by the ACSM guidelines yield significantly greater benefits in improving quality of life, alleviating cancer-related fatigue, and controlling pain in patients with lung cancer. These findings have clear clinical relevance and provide robust evidence to support the development of standardized and structured exercise interventions in lung cancer rehabilitation. Accordingly, we recommend that clinicians initiate individualized exercise programs for patients with lung cancer as early as feasible, and, while ensuring safety, progressively tailor and optimize exercise parameters based on patients’ functional status, treatment phase, and tolerance to achieve high adherence to the ACSM guidelines. In practice, personalized exercise prescriptions can be implemented within the ACSM framework, with particular emphasis on the concurrent management of key symptoms such as fatigue and pain. Future research should prioritize large-scale, multicenter, high-quality randomized controlled trials that rigorously adhere to the ACSM guidelines to further validate these findings and facilitate the establishment and refinement of systematic exercise-based rehabilitation models for lung cancer.

Reflecting on the methodological constraints introduced earlier, the limitations of this study primarily center on the following aspects. First, although adherence categories were established based on the ACSM guidelines, substantial heterogeneity existed across the included studies with respect to specific exercise parameters, including exercise modality, intensity, and frequency. The scoring methodology itself also presents a potential source of misclassification bias. While assigning a neutral weight of 1 point to parameters deemed ‘unable to determine’ aligns with established research practices, this approach may still lead to imprecise adherence estimates in certain trials. Accordingly, comparisons between high and low/uncertain adherence subgroups should be interpreted with caution, as these findings are fundamentally constrained by the reporting quality of the primary literature. Second, considerable heterogeneity was also present in patient characteristics. Specifically, participants across studies were at different stages of lung cancer treatment, and varied in pathological subtype, disease stage, and prior treatment regimens. While this clinical and pathological diversity reflects real-world practice to some extent, it may introduce significant confounding, thereby affecting the reliability of effect estimates and limiting the certainty of the conclusions. The persistent heterogeneity in certain outcomes, particularly within the high-adherence subgroup for Pain (*I²* = 95%), warrants careful consideration. This variance likely stems from both methodological factors, such as the use of inconsistent assessment tools, and clinical factors, including the multifaceted etiology of lung cancer pain (e.g., post-operative pain vs. cancer-related bone pain). Furthermore, broader clinical variations, such as diverse tumor stages and treatment phases (e.g., concurrent chemotherapy vs. post-surgical recovery), may further contribute to the overall variance. However, as most included studies encompassed mixed patient cohorts with overlapping stages or failed to report granular clinical characteristics, performing independent stratified analyses remains challenging. This lack of patient-specific data in primary studies remains a fundamental limitation in resolving the residual heterogeneity. Third, a methodological limitation exists in our subgroup analysis regarding intervention adherence. Due to our scoring framework, studies often exhibit a mixture of confirmed ‘low-dose’ parameters and ‘insufficiently reported’ dimensions, which were pooled into a single ‘low or uncertain’ category. This grouping strategy may inadvertently lead to the dilution or exaggeration of true subgroup differences by confounding genuine low efficacy with under-reporting in the primary literature. Consequently, the lack of statistically significant benefits observed in this subgroup should be interpreted with caution, as it may reflect either a true physiological lack of effect or a lack of descriptive clarity in the original trials. Moreover, incomplete or nonstandardized reporting of exercise prescriptions was observed in several studies, particularly regarding intensity monitoring methods, individualized progression strategies, and actual completion rates, which may have led to misclassification of adherence and introduced measurement bias, potentially resulting in some high-quality interventions being inaccurately categorized as having low or uncertain adherence. A primary limitation of the current evidence base is the structural impossibility of blinding participants and personnel in exercise interventions. Under the RoB 2 framework, this constraint necessitated a rating of ‘some concerns’ for deviations from intended interventions (Domain 2) and, more critically, a ‘high risk’ for outcome measurement (Domain 4) given the subjective nature of the assessment scales. Additionally, approximately 25% of the trials raised concerns regarding the randomization process due to incomplete reporting of concealment procedures. These factors, combined with the observed statistical heterogeneity, ultimately dictated the ‘overall high risk’ assessment and the ‘low’ to ‘very low’ certainty of evidence across all outcomes. Another important consideration is that adherence classification in this study was entirely based on the ACSM guidelines, which were originally developed for healthy populations. Patients with lung cancer frequently experience substantial physical deconditioning, treatment-related adverse effects, and comorbidities, and their optimal exercise dose may therefore differ from guideline recommendations. Consequently, while the adherence framework used here is informative, it may have theoretical limitations in fully capturing the “truly appropriate” exercise dose for this population. Additionally, key outcomes such as quality of life, fatigue, depression, and anxiety were primarily assessed using patient-reported instruments, and differences in measurement tools across studies (e.g., EORTC QLQ-C30, SF-36, FACT-L) in terms of dimensional structure, sensitivity, and cultural adaptation may have introduced measurement heterogeneity and affected cross-study comparability. Moreover, beyond individual study bias, our GRADE assessment underscores a critical gap between statistical significance and the certainty of evidence. Although most health-related outcomes achieved a *p*-value < 0.05, the ‘low’ to ‘very low’ certainty suggests that our confidence in these effect estimates is limited, and the true effects may be substantially different. This uncertainty stems from the cumulative impact of serious inconsistency and imprecision across the included trials, particularly for pain and sleep quality where sample sizes often fell below the optimal information size. Consequently, while our findings suggest that exercise is a promising supportive intervention for lung cancer patients, they should be viewed as preliminary clinical evidence. Furthermore, the assessment of publication bias in this meta-analysis is subject to certain methodological limitations. While funnel plots and Egger’s tests did not reveal significant evidence of bias for most outcomes, the statistical power of these assessments was inherently constrained by the limited number of included trials for certain indicators (e.g., pain, n=9) and the overall high statistical heterogeneity. Consequently, the absence of statistically significant results in these tests does not definitively exclude the possibility of publication bias, and these findings should be interpreted as exploratory rather than confirmatory. In light of these findings and limitations, future research should prioritize several directions: first, large-scale, stratified clinical studies specifically targeting patients with lung cancer are needed to determine safe and effective exercise parameters tailored to different pathological subtypes, disease stages, and treatment phases, thereby informing lung cancer–specific exercise rehabilitation guidelines; second, more methodologically rigorous RCTs should be conducted, with improved allocation concealment, implementation of blinded outcome assessment, and standardized reporting of exercise supervision and adherence data to enhance the strength of the evidence; third, further investigation into the biological and psychosocial mechanisms underlying exercise-induced improvements in quality of life and symptom burden is warranted, alongside the adoption of unified, validated core outcome sets to improve comparability and interpretability across studies; finally, implementation science approaches should be employed to explore how effective, individualized exercise interventions can be integrated into routine clinical pathways, thereby facilitating the development of scalable and sustainable exercise-based rehabilitation systems for lung cancer.

## 5 Conclusions

This meta-analysis suggests that exercise interventions with high adherence to the ACSM guidelines may confer more consistent benefits in improving quality of life and reducing fatigue and pain in patients with lung cancer. In contrast, no significant additional advantages of high-adherence exercise over low or uncertain-adherence interventions were observed for alleviating anxiety and depression, which may be attributable to the relatively small number of included studies, substantial sample heterogeneity, and the presence of uncontrolled confounding factors. Notably, the findings for sleep quality should not be interpreted as a mere lack of effect; rather, a clear dose-related trend was observed, with high-adherence exercise demonstrating borderline significance and the difference between levels approaching significance. These findings, however, should be interpreted with caution due to the pervasive risk of bias and limited statistical power from small sample sizes. Therefore, future research should focus on large-scale, high-quality prospective studies to further elucidate the effects of exercise interventions with varying levels of adherence on multidimensional health outcomes in patients with lung cancer, thereby providing more robust and reliable evidence to inform clinical practice.

## Data Availability

The original contributions presented in the study are included in the article/**Supplementary Material**. Further inquiries can be directed to the corresponding authors.
