# Supplementary material for: Effects of ACSM guideline–based exercise on patients with lung cancer: a systematic review and meta-analysis
Source: Front Physiol. 2026 Apr 15;17:1797432. doi: 10.3389/fphys.2026.1797432 (PMC13126151; doi:10.3389/fphys.2026.1797432)
Supplement: Supplementary file 1 [file SupplementaryFile1.docx]

| Database | Search strategy |
| --- | --- |
|  | Pubmed |
| #1 | Lung Neoplasms[MeSH Terms] 300184 |
| #2 | (((((((((((((((((Lung Neoplasms[Title/Abstract]) OR (Neoplasms, Pulmonary[Title/Abstract])) OR (Neoplasm, Pulmonary[Title/Abstract])) OR (Pulmonary Neoplasm[Title/Abstract])) OR (Pulmonary Neoplasms[Title/Abstract])) OR (Neoplasms, Lung[Title/Abstract])) OR (Lung Neoplasm[Title/Abstract])) OR (Neoplasm, Lung[Title/Abstract])) OR (Lung Cancer[Title/Abstract])) OR (Cancer, Lung[Title/Abstract])) OR (Cancers, Lung[Title/Abstract])) OR (Lung Cancers[Title/Abstract])) OR (Cancer of Lung[Title/Abstract])) OR (Pulmonary Cancer[Title/Abstract])) OR (Cancer, Pulmonary[Title/Abstract])) OR (Cancers, Pulmonary[Title/Abstract])) OR (Pulmonary Cancers[Title/Abstract])) OR (Cancer of the Lung[Title/Abstract]) 250901 |
| #3 | (#1) OR (#2) 383587 |
| #4 | (Exercise[MeSH Terms]) OR (Circuit-Based Exercise[MeSH Terms]) 276663 |
| #5 | ((((((((((((((((((((((((((((((((Exercise[Title/Abstract]) OR (Circuit-Based Exercise[Title/Abstract])) OR (Exercises[Title/Abstract])) OR (Exercise, Physical[Title/Abstract])) OR (Exercises, Physical[Title/Abstract])) OR (Physical Exercise[Title/Abstract])) OR (Physical Exercises[Title/Abstract])) OR (Exercise, Aerobic[Title/Abstract])) OR (Aerobic Exercise[Title/Abstract])) OR (Aerobic Exercises[Title/Abstract])) OR (Exercises, Aerobic[Title/Abstract])) OR (Exercise, Isometric[Title/Abstract])) OR (Exercises, Isometric[Title/Abstract])) OR (Isometric Exercises[Title/Abstract])) OR (Isometric Exercise[Title/Abstract])) OR (Acute Exercise[Title/Abstract])) OR (Acute Exercises[Title/Abstract])) OR (Exercise, Acute[Title/Abstract])) OR (Exercises, Acute[Title/Abstract])) OR (Exercise Training[Title/Abstract])) OR (Exercise Trainings[Title/Abstract])) OR (Training, Exercise[Title/Abstract])) OR (Trainings, Exercise[Title/Abstract])) OR (Physical Activity[Title/Abstract])) OR (Activities, Physical[Title/Abstract])) OR (Activity, Physical[Title/Abstract])) OR (Physical Activities[Title/Abstract])) OR (Circuit Based Exercise[Title/Abstract])) OR (Circuit-Based Exercises[Title/Abstract])) OR (Exercise, Circuit-Based[Title/Abstract])) OR (Exercises, Circuit-Based[Title/Abstract])) OR (Circuit Training[Title/Abstract])) OR (Training, Circuit[Title/Abstract]) 536313 |
| #6 | (#4) OR (#5) 633163 |
| #7 | (#3) AND (#6) 2283 |
|  | Embase |
| #1 | 'lung neoplasms':ab,ti OR 'neoplasms, pulmonary':ab,ti OR 'neoplasm, pulmonary':ab,ti OR 'pulmonary neoplasm':ab,ti OR 'pulmonary neoplasms':ab,ti OR 'neoplasms, lung':ab,ti OR 'lung neoplasm':ab,ti OR 'neoplasm, lung':ab,ti OR 'lung cancer':ab,ti OR 'cancer, lung':ab,ti OR 'cancers, lung':ab,ti OR 'lung cancers':ab,ti OR 'cancer of lung':ab,ti OR 'pulmonary cancer':ab,ti OR 'cancer, pulmonary':ab,ti OR 'cancers, pulmonary':ab,ti OR 'pulmonary cancers':ab,ti  375669 |
| #2 | exercise:ab,ti OR 'circuit-based exercise':ab,ti OR exercises:ab,ti OR 'exercise, physical':ab,ti OR 'exercises, physical':ab,ti OR 'physical exercise':ab,ti OR 'physical exercises':ab,ti OR 'exercise, aerobic':ab,ti OR 'aerobic exercise':ab,ti OR 'aerobic exercises':ab,ti OR 'exercises, aerobic':ab,ti OR 'exercise, isometric':ab,ti OR 'exercises, isometric':ab,ti OR 'isometric exercises':ab,ti OR 'isometric exercise':ab,ti OR 'acute exercise':ab,ti OR 'acute exercises':ab,ti OR 'exercise, acute':ab,ti OR 'exercises, acute':ab,ti OR 'exercise training':ab,ti OR 'exercise trainings':ab,ti OR 'training, exercise':ab,ti OR 'trainings, exercise':ab,ti OR 'physical activity':ab,ti OR 'activities, physical':ab,ti OR 'activity, physical':ab,ti OR 'physical activities':ab,ti OR 'circuit based exercise':ab,ti OR 'circuit-based exercises':ab,ti OR 'exercise, circuit-based':ab,ti OR 'exercises, circuit-based':ab,ti OR 'circuit training':ab,ti OR 'training, circuit':ab,ti  746155 |
| #3 | #1 AND #2  3034 |
|  | Web of Science |
| #1 | Lung Neoplasms (Topic) or Neoplasms, Pulmonary (Topic) or Neoplasm, Pulmonary (Topic) or Pulmonary Neoplasm (Topic) or Pulmonary Neoplasms (Topic) or Neoplasms, Lung (Topic) or Lung Neoplasm (Topic) or Neoplasm, Lung (Topic) or Lung Cancer (Topic) or Cancer, Lung (Topic) or Cancers, Lung (Topic) or Lung Cancers (Topic) or Cancer of Lung (Topic) or Pulmonary Cancer (Topic) or Cancer, Pulmonary (Topic) or Cancers, Pulmonary (Topic) or Pulmonary Cancers (Topic)  508240 |
| #2 | Exercise (Topic) or Circuit-Based Exercise (Topic) or Exercises (Topic) or Exercise, Physical (Topic) or Exercises, Physical (Topic) or Physical Exercise (Topic) or Physical Exercises (Topic) or Exercise, Aerobic (Topic) or Aerobic Exercise (Topic) or Aerobic Exercises (Topic) or Exercises, Aerobic (Topic) or Exercise, Isometric (Topic) or Exercises, Isometric (Topic) or Isometric Exercises (Topic) or Isometric Exercise (Topic) or Acute Exercise (Topic) or Acute Exercises (Topic) or Exercise, Acute (Topic) or Exercises, Acute (Topic) or Exercise Training (Topic) or Exercise Trainings (Topic) or Training, Exercise (Topic) or Trainings, Exercise (Topic) or Physical Activity (Topic) or Activities, Physical (Topic) or Activity, Physical (Topic) or Physical Activities (Topic) or Circuit Based Exercise (Topic) or Circuit-Based Exercises (Topic) or Exercise, Circuit-Based (Topic) or Exercises, Circuit-Based (Topic) or Circuit Training (Topic) or Training, Circuit (Topic)  1025480 |
| #3 | #1 AND #2  5553 |
|  | Cochrane |
| #1 | (Lung Neoplasms):ti,ab,kw OR (Neoplasms, Pulmonary):ti,ab,kw OR (Neoplasm, Pulmonary):ti,ab,kw OR (Pulmonary Neoplasm):ti,ab,kw OR (Pulmonary Neoplasms):ti,ab,kw 17762 |
| #2 | (Neoplasms, Lung):ti,ab,kw OR (Lung Neoplasm):ti,ab,kw OR (Neoplasm, Lung):ti,ab,kw OR (Lung Cancer):ti,ab,kw OR (Cancer, Lung):ti,ab,kw 36033 |
| #3 | (Cancers, Lung):ti,ab,kw OR (Lung Cancers):ti,ab,kw OR (Cancer of Lung):ti,ab,kw OR (Pulmonary Cancer):ti,ab,kw OR (Cancer, Pulmonary):ti,ab,kw 35476 |
| #4 | (Cancers, Pulmonary):ti,ab,kw OR (Pulmonary Cancers):ti,ab,kw 5045 |
| #5 | #1 OR #2 OR #3 OR #4  37705 |
| #6 | (Exercise):ti,ab,kw OR (Circuit-Based Exercise):ti,ab,kw OR (Exercises):ti,ab,kw OR (Exercise, Physical):ti,ab,kw OR (Exercises, Physical):ti,ab,kw  162126 |
| #7 | (Physical Exercise):ti,ab,kw OR (Physical Exercises):ti,ab,kw OR (Exercise, Aerobic):ti,ab,kw OR (Aerobic Exercise):ti,ab,kw OR (Aerobic Exercises):ti,ab,kw  75780 |
| #8 | (Exercises, Aerobic):ti,ab,kw OR (Exercise, Isometric):ti,ab,kw OR (Exercises, Isometric):ti,ab,kw OR (Isometric Exercises):ti,ab,kw OR (Isometric Exercise):ti,ab,kw  26133 |
| #9 | (Acute Exercise):ti,ab,kw OR (Acute Exercises):ti,ab,kw OR (Exercise, Acute):ti,ab,kw OR (Exercises, Acute):ti,ab,kw OR (Exercise Training):ti,ab,kw  70679 |
| #10 | (Exercise Trainings):ti,ab,kw OR (Training, Exercise):ti,ab,kw OR (Trainings, Exercise):ti,ab,kw OR (Activities, Physical):ti,ab,kw OR (Activity, Physical):ti,ab,kw  131850 |
| #11 | (Physical Activities):ti,ab,kw OR (Circuit Based Exercise):ti,ab,kw OR (Circuit-Based Exercises):ti,ab,kw OR (Exercise, Circuit-Based):ti,ab,kw OR (Exercises, Circuit-Based):ti,ab,kw  86614 |
| #12 | (Circuit Training):ti,ab,kw OR (Training, Circuit):ti,ab,kw  1638 |
| #13 | #6 OR #7 OR #8 OR #9 OR #10 OR #11 OR #12  209496 |
| #14 | #5 AND #13 1603 |
