# Supplementary material for: Effects of ACSM guideline–based exercise on patients with lung cancer: a systematic review and meta-analysis
Source: Front Physiol. 2026 Apr 15;17:1797432. doi: 10.3389/fphys.2026.1797432 (PMC13126151; doi:10.3389/fphys.2026.1797432)
Supplement: Supplementary file 2 [file Table1.docx]

**Supplementary Table 1 ACSM exercise recommendations**

| Exercise dose | Cardiorespiratory exercise | Resistance exercise | Flexibility exercise |
| --- | --- | --- | --- |
| Frequency | 3–5 days per week | 2–3 days per week | ≥ 2–3 days per week |
| Intensity/workload | 40–60% VO^2^R or HRR; RPE of 12–13 on a 6–20 scale;55–90% of maximal heart rate | Start with 40%–50% 1RM, more capable with 60%–70% 1RM | Stretch until you feel your muscles being pulled tight or a slight discomfort |
| Duration | Continuous or cumulative 30 min | Starting with one set of 8–12 repetitions, increase to two sets after about 2 weeks. Perform no more than 8–10 exercises per session | Static stretching held for 10–30 s, repeated 2–4 times |

HRR heart rate reserve, VO^2^R oxygen uptake reserve, RPE rating of perceived exertion, 1RM one repetition maximum
