# Supplementary material for: Effects of ACSM guideline–based exercise on patients with lung cancer: a systematic review and meta-analysis
Source: Front Physiol. 2026 Apr 15;17:1797432. doi: 10.3389/fphys.2026.1797432 (PMC13126151; doi:10.3389/fphys.2026.1797432)
Supplement: Supplementary file 3 [file Table2.docx]

**Supplementary Table 2 Study characteristics**

| Author | Country | Year | Participants | Age(years) | | Total/male/female | | Interventions | Length of intervention | Control | Outcome |
| --- | --- | --- | --- | --- | --- | --- | --- | --- | --- | --- | --- |
|  |  |  |  | IG | CG | IG | CG |  |  |  |  |
| Arbane, Gill et al. | United Kingdom | 2011 | NSCLC | 62.60 ± 11.11 | 65.40 ± 25.93 | 26 | 25 | Individualised Home-Based Exercise Programme | 12 weeks | Usual care | Quality of life |
| Bade, Brett C et al. | United States | 2021 | III/IV NSCLC | 66.55 ± 7.28 | 63.20 ± 9.80 | 20/8/12 | 20/2/18 | Individualized Walking Goals  Freq: 7 times a week | 12 weeks | Usual care | Quality of life  Fatigue  Pain  Insomnia |
| Bloch, Zina et al. | Denmark | 2025 | IIIb/IV SCLC or  NSCLC | 65.00 ± 8.00 | 64.00 ± 9.00 | 110/55/55 | 108/52/56 | Aerobic training  Strength training  Flexibility training  Freq: 2 times a week  Duration:90 mins | 12 weeks | Usual care | Quality of life  Fatigue  Pain  Insomnia |
| Lee, Jang Ho et al. | Korea | 2025 | NSCLC/SCLC | 60.30 ± 13.10 | 62.60 ± 10.00 | 33/23/10 | 32/21/11 | Aerobic training  Strength training  Freq: 2-3 times a week  Duration:60 mins | 4-6 weeks | Usual care | Quality of life |
| Messaggi-Sartor, Monique et al. | Spain | 2019 | I/II NSCLC | 64.20 ± 8.10 | 64.80 ± 8.90 | 16/8/8 | 21/18/3 | Aerobic training  Strength training  Freq: 3 times a week  Duration:60 mins | 8 weeks | Usual care | Quality of life  Fatigue  Pain |
| Wang,Yan-Li et al. | China | 2024 | II-IV NSCLC | 54.44 ± 8.07 | 54.40 ± 9.96 | 30/20/10 | 30/20/10 | Strength training  Flexibility training  Freq: 3-7 times a week | 8 weeks | Usual care | depression  anxiety  Insomnia |
| Xu, Jilai et al. | China | 2024 | I-IIIA NSCLC | NR | NR | 108 | 108 | Baduanjin  Freq: 4 times a week  Duration:30 mins | 12 weeks | Usual care | Fatigue |
| Chen, Hui-Mei et al. | China | 2016 | I-IV Lung cancer | 64.64 ± 11.54 | 62.51 ± 9.64 | 56/24/32 | 55/25/30 | Home-based walking exercise  Freq: 3 times a week  Duration:40 mins | 12 weeks | Usual care | Insomnia |
| Quist, Morten et al. | Denmark | 2020 | IIIb/IV NSCLC or  ED-SCLC | 65.20 ± 8.20 | 63.50 ± 8.70 | 110/55/55 | 108/52/56 | Aerobic training  Strength training  Flexibility training  Freq: 2 times a week  Duration:90 mins | 12 weeks | Usual care | Quality of life  depression  anxiety |
| Hwang, Chueh-Lung et al. | China | 2012 | IIIB/IV NSCLC | 61.00 ± 6.30 | 58.50 ± 8.20 | 13/5/8 | 11/7/4 | Aerobic training  Freq: 3 times a week  Duration:30-40 mins | 8 weeks | Usual care | Quality of life  Fatigue |
| Cavalheri, Vinicius et al. | Australia | 2017 | I-IIIA NSCLC | 66.00 ± 10.00 | 68.00 ± 9.00 | 9/3/6 | 8/2/6 | Aerobic training  Strength training  Freq: 3 times a week  Duration:60 mins | 8 weeks | Usual care | Quality of life  Fatigue  depression  anxiety |
| Cheung, Denise Shuk Ting et al. Tai Chi | China | 2021 | IIIB/IV NSCLC | 61.11 ± 7.01 | 58.36 ± 9.32 | 9/6/3 | 11/5/6 | Tai-chi  Freq: 5 times a week  Duration:30-60 mins | 12 weeks | Health education | Quality of life  Fatigue  depression  anxiety  Insomnia |
| Cheung, Denise Shuk Ting et al. Aerobic | China | 2021 | IIIB/IV NSCLC | 61.00 ± 12.12 | 58.36 ± 9.32 | 10/5/5 | 11/5/6 | Aerobic training  Strength training  Freq: 3-5 times a week  Duration:60-90 mins | 12 weeks | Health education | Quality of life  Fatigue  depression  anxiety  Insomnia |
| Granger, Catherine L et al. | Australia | 2024 | I-IIIA NSCLC | 65.40 ± 10.80 | 67.50 ±8.10 | 58/25/33 | 58/23/35 | Aerobic training  Strength training  Individualised | 3 months | Usual care | Quality of life  Fatigue  Pain  Insomnia |
| Dhillon, H.M. et al. | Australia | 2017 | III/IV NSCLC/SCLC | 64.00±31.11 | 64.00±31.11 | 56/29/27 | 55/32/23 | Aerobic training  Strength training  Freq: 1 times a week | 8 weeks | Usual care | Quality of life  Fatigue  Insomnia |
| Jonsson, Marcus et al. | Sweden | 2019 | NSCLC | 68.70 ± 7.40 | 68.40 ± 8.30 | 54/29/25 | 53/18/35 | Flexibility training  Freq: 7 times a week | 1 week | Usual care | Pain |
| Ma, Rui-Chen et al. | China | 2021 | I-IIIA  NSCLC | 56.97 ± 7.09 | 54.91 ± 10.09 | 34/13/21 | 35/8/27 | Aerobic training  Freq: 7 times a week  Duration:70 mins | 2 weeks | Usual care | depression  anxiety |
| Ulrich,Cornelia M. et al. | United States | 2025 | I-IIIA  Lung cancer | 62.70±14.70 | 62.70±13.00 | 92/51/41 | 90/57/33 | Aerobic training  Strength training  Flexibility training  Individualised | 2 months | Usual care | Fatigue |
| Zhou, Nanjiang et al. | China | 2025 | I-II  NSCLC | 57.00 ±11.11 | 56.00 ±6.67 | 51/19/32 | 50/21/29 | Aerobic training  Strength training  Flexibility training  Freq: 3-4 times a week  Duration:40 mins | 16 days | Usual care | Quality of life  depression  anxiety |
| Egegaard, Trine et al. | Denmark | 2019 | IIIA/IIIB NSCLC | 64.00 ± 5.80 | 65.00 ± 4.70 | 8/3/5 | 7/2/5 | Aerobic training  Freq: 5 times a week  Duration:20 mins | 7 weeks | Usual care | Quality of life  depression  anxiety |
| Chen, H-M et al. | China | 2015 | I-IV Lung cancer | 64.76±11.28 | 63.57±10.54 | 58/26/32 | 58/28/30 | Walking-exercise  Freq: 3 times a week  Duration:40 mins | 12 weeks | Usual care | depression  anxiety |
| Wu, Jing et al. | China | 2025 | NSCLC/SCLC | 66.29 ± 3.81 | 66.38 ± 4.01 | 31/20/11 | 32/18/14 | Baduanjin  Freq: 3 times a week  Duration:50 mins | 12 weeks | Usual care | Fatigue  Insomnia |
| Lai, Yutian et al. | China | 2017 | I-IV NSCLC | 63.80 ± 8.20 | 64.60 ± 6.60 | 51/28/23 | 50/37/13 | Aerobic training  Freq: 7 times a week  Duration:30 mins | 7 days | Usual care | Quality of life |
| Henke, C.C. et al. | Germany | 2014 | IIIA/IIIB/IV  NSCLC/SCLC | NR | NR | 23 | 23 | Aerobic training  Freq: 5 times a week  Strength training  Freq: 3 times a week | NR | Usual care | Quality of life  Fatigue  Insomnia |
| Ha, Duc M. et al. | United States | 2023 | 1. IIIA NSCLC/SCLC | 68.30 ± 6.40 | 70.30 ± 7.40 | 14/8/6 | 14/7/7 | Aerobic training  Freq: 4-5 times a week | 12 weeks | Health education | Quality of life  Insomnia |
| Turan, Gülcan Bahcecioglu et al. | Turkey | 2024 | I-IV Lung cancer | 60.08 ± 11.99 | 63.14 ± 7.79 | 37/23/14 | 37/21/16 | Flexibility training  Freq: 7 times a week  Duration:30 mins | 8 weeks | Usual care | Pain  Insomnia |
| Liu, Zijia et al. | China | 2020 | I-III NSCLC | 56.20±10.30 | 56.20 ±8.70 | 37/12/25 | 36/11/25 | Aerobic training  Freq: 3 times a week  Strength training  Freq: 2 times a week | 2 weeks | Usual care | depression  anxiety |
| Molassiotis, Alex et al. | Vietnam | 2021 | I-IV NSCLC/SCLC | 57.62 ±9.63 | 56.06 ±9.25 | 78/59/19 | 78/57/21 | Qigong  Freq: 5 times a week  Duration:30 mins | 6 weeks | Usual care | Quality of life  Fatigue  anxiety |
| Rehman, Muheebur et al. | Pakistan | 2023 | I-II NSCLC | 48.10 ±4.00 | 48.30 ±3.80 | 20/11/9 | 20/13/7 | Aerobic training  Freq: 5 times a week  Duration:20 mins | 4 weeks | Usual care | depression  Anxiety  Pain |
| Morano, Maria Tereza Aguiar Pessoa et al. | Brazil | 2014 | I-IIIA NSCLC | 65.00 ± 8.00 | 69.00 ± 7.00 | 12/4/8 | 12/5/7 | Aerobic training  Strength training  Flexibility training  Freq: 5 times a week | 4 weeks | Usual care | depression  Anxiety |
| Sui, Yiling et al. | China | 2020 | I-III NSCLC | 61.37±11.21 | 62.35±9.98 | 100/80/20 | 100/84/16 | Individualized Walking Goals | 12 months | Usual care | Quality of life  depression  Anxiety |
| Jiang, Mi et al. | China | 2020 | NSCLC | 59.30 ± 7.40 | 57.56 ± 11.23 | 50/27/23 | 50/29/21 | Tai Chi  Freq: 5 times a week  Duration:40-60 mins | 3 months | Usual care | Pain |

Note: Numbers are mean ± SD unless otherwise stated. NR,not report; IG,Intervention group; CG,Control group;SCLC, small cell lung cancer; NSCLC, non-small cell lung cancer.
