# Supplementary material for: Effects of ACSM guideline–based exercise on patients with lung cancer: a systematic review and meta-analysis
Source: Front Physiol. 2026 Apr 15;17:1797432. doi: 10.3389/fphys.2026.1797432 (PMC13126151; doi:10.3389/fphys.2026.1797432)
Supplement: Supplementary file 4 [file Table3.docx]

Supplementary Table 3 Exercise interventions evaluated according to the American College of Sports Medicine (ACSM) recommendations

| Author, year | Cardiorespiratory exercise | | | | | | Resistance exercise | | | | | | | | Flexibility exercise | | | | | | ACSM compliance | |
| --- | --- | --- | --- | --- | --- | --- | --- | --- | --- | --- | --- | --- | --- | --- | --- | --- | --- | --- | --- | --- | --- | --- |
|  | Frequency  Days/week | | Intensity/  workload | | Duration  (min) | | Frequency  Days/week | | Intensity/  workload | | Repetition  (min) | | Set  (group) | | Frequency  Days/week | | Intensity/  workload | | Duration  (min) | | Points/  Percent | |
| Arbane, Gill et al. (2011) | NR | 😐 | NR | 😐 | NR | 😐 | NR | 😐 | NR | 😐 | NR | 😐 | NR | 😐 |  |  |  |  |  |  | 7/14 | 50% |
| Bade, Brett C et al. (2021) | 7 | ☺ | NR | 😐 | NR | 😐 |  |  |  |  |  |  |  |  |  |  |  |  |  |  | 4/6 | 67% |
| Bloch, Zina et al. (2025) | 2 | 😐 | 70-90% MHR | ☺ | 20-25 | 😐 | 2 | ☺ | 70–90% 1RM | 😐 | 5-8 | 😐 | 3 | ☺ | 2 | ☺ | NR | 😐 | 5-10 | ☺ | 15/20 | 75% |
| Lee, Jang Ho et al.(2025) | 2-3 | 😐 | RPE 13-15 | ☺ | 20 | 😐 | 2-3 | ☺ | NR | 😐 | NR | 😐 | NR | 😐 |  |  |  |  |  |  | 9/14 | 64% |
| Messaggi-Sartor, Monique et al. (2019) | 3 | ☺ | NR | 😐 | 30 | ☺ | 3 | ☺ | NR | 😐 | NR | 😐 | NR | 😐 |  |  |  |  |  |  | 10/14 | 71% |
| Wang,Yan-Li et al. (2024) |  |  |  |  |  |  | 3-7 | 😐 | NR | 😐 | 10-12 | ☺ | 3 | ☺ | 7 | ☺ | NR | 😐 | 10-15 | ☺ | 11/14 | 79% |
| Xu, Jilai et al. (2024) | 4 | ☺ | NR | 😐 | 60 | ☺ |  |  |  |  |  |  |  |  | 4 | ☺ | NR | 😐 | NR | 😐 | 9/12 | 75% |
| Chen, Hui-Mei et al.( 2016) | 3 | ☺ | RPE of 13-15 | ☺ | 40 | ☺ |  |  |  |  |  |  |  |  |  |  |  |  |  |  | 6/6 | 100% |
| Quist, Morten et al. (2020) | 2 | 😐 | 70-90% MHR | ☺ | 20-25 | 😐 | 2 | ☺ | 70–90% 1RM | 😐 | 5-8 | 😐 | 3 | ☺ | 2 | ☺ | NR | 😐 | 5-10 | ☺ | 15/20 | 75% |
| Hwang, Chueh-Lung et al. (2012) | 3 | ☺ | 60-80%VO2max | 😐 | 30-40 | ☺ |  |  |  |  |  |  |  |  |  |  |  |  |  |  | 5/6 | 83% |
| Cavalheri, Vinicius et al. (2017) | 3 | ☺ | NR | 😐 | 30 | ☺ | 3 | ☺ | NR | 😐 | 10 | ☺ | 2 | ☺ |  |  |  |  |  |  | 12/14 | 86% |
| Cheung, Denise Shuk Ting et al. Tai Chi (2021) | 5 | ☺ | NR | 😐 | 30-60 | ☺ |  |  |  |  |  |  |  |  | 5 | ☺ | NR | 😐 | NR | 😐 | 9/12 | 75% |
| Cheung, Denise Shuk Ting et al. Aerobic (2021) | 3-5 | ☺ | 50-60% HHR | ☺ | 30 | ☺ | 2 | ☺ | 60% 1RM | ☺ | 10 | ☺ | NR | 😐 |  |  |  |  |  |  | 13/14 | 93% |
| Granger, Catherine L et al. (2024) | NR | 😐 | NR | 😐 | NR | 😐 | NR | 😐 | NR | 😐 | NR | 😐 | NR | 😐 |  |  |  |  |  |  | 7/14 | 50% |
| Dhillon, H.M. et al. (2017) | 1 | ☹ | NR | 😐 | 45 | ☺ |  |  |  |  |  |  |  |  |  |  |  |  |  |  | 3/6 | 50% |
| Jonsson, Marcus et al. (2019) |  |  |  |  |  |  |  |  |  |  |  |  |  |  | 7 | ☺ | NR | 😐 | NR | 😐 | 4/6 | 67% |
| Ma, Rui-Chen et al. (2021) | 7 | ☺ | 70% MHR | ☺ | 10-20 | ☹ |  |  |  |  |  |  |  |  |  |  |  |  |  |  | 4/6 | 67% |
| Ulrich,Cornelia M. et al. (2025) | NR | 😐 | NR | 😐 | 5-30 | 😐 | NR | 😐 | NR | 😐 | NR | 😐 | NR | 😐 | NR | 😐 | NR | 😐 | NR | 😐 | 10/20 | 50% |
| Zhou, Nanjiang et al.(2025) | 3-4 | ☺ | NR | 😐 | 30 | ☺ | 3-4 | ☺ | NR | 😐 | NR | 😐 | NR | 😐 | 3-4 | ☺ | NR | 😐 | NR | 😐 | 14/20 | 70% |
| Egegaard, Trine et al.(2019) | 5 | ☺ | NR | 😐 | 20 | 😐 |  |  |  |  |  |  |  |  |  |  |  |  |  |  | 4/6 | 67% |
| Chen, H-M et al.(2015) | 3 | ☺ | 60-80% HHR | 😐 | 40 | ☺ |  |  |  |  |  |  |  |  |  |  |  |  |  |  | 5/6 | 83% |
| Wu, Jing et al.(2025) | 3 | ☺ | NR | 😐 | 40 | ☺ |  |  |  |  |  |  |  |  | 3 | ☺ | NR | 😐 | NR | 😐 | 9/12 | 75% |
| Lai, Yutian et al.(2017) | 7 | ☺ | NR | 😐 | 30 | ☺ |  |  |  |  |  |  |  |  |  |  |  |  |  |  | 5/6 | 83% |
| Henke, C.C. et al.(2014） | 5 | ☺ | 55-70% HHR | 😐 | 8 | ☹ | 3 | ☺ | 50% 1RM | ☺ | 10 | ☺ | 3 | ☺ |  |  |  |  |  |  | 11/14 | 79% |
| Ha, Duc M. et al.(2023) | 4-5 | ☺ | NR | 😐 | NR | 😐 |  |  |  |  |  |  |  |  |  |  |  |  |  |  | 4/6 | 67% |
| Turan, Gülcan Bahcecioglu et al.(2024） |  |  |  |  |  |  |  |  |  |  |  |  |  |  | 7 | ☺ | NR | 😐 | 30 | ☺ | 5/6 | 83% |
| Liu, Zijia et al.（2020） | 3 | ☺ | RPE of 13–16 | 😐 | 25 | 😐 | 2 | ☺ | NR | 😐 | 10-12 | ☺ | 3 | ☺ |  |  |  |  |  |  | 12/14 | 86% |
| Molassiotis, Alex et al.（2021） | 5 | ☺ | NR | 😐 | 30-90 | ☺ |  |  |  |  |  |  |  |  | 5 | ☺ | NR | 😐 | 30 | ☺ | 10/12 | 83% |
| Rehman, Muheebur et al. （2023） | 5 | ☺ | 40-60% HHR | ☺ | 20 | 😐 |  |  |  |  |  |  |  |  |  |  |  |  |  |  | 5/6 | 83% |
| Morano, Maria Tereza Aguiar Pessoa et al.(2014) | 5 | ☺ | 80%VO2max | ☹ | NR | 😐 | 5 | ☺ | 50% 1RM | ☺ | NR | 😐 | NR | 😐 | 5 | ☺ | NR | 😐 | NR | 😐 | 13/20 | 65% |
| Sui, Yiling et al. （2020） | 7 | ☺ | NR | 😐 | NR | 😐 | 1 | ☹ | NR | 😐 | NR | 😐 | NR | 😐 | 1 | ☹ | NR | 😐 | NR | 😐 | 9/20 | 45% |
| Jiang, Mi et al.（2020） | 5 | ☺ | NR | 😐 | 40 | ☺ |  |  |  |  |  |  |  |  | 5 | ☺ | NR | 😐 | 40 | ☺ | 10/12 | 83% |

ACSM, American College of Sports Medicine. NR, not reported. Happy/green face, fulfils recommendation (2 points), neutral/yellow face, unable to determine due to insufficient reporting (1 point), unhappy/red face, does not fulfil recommendation (0 point).
