# Supplementary material for: Effects of ACSM guideline–based exercise on patients with lung cancer: a systematic review and meta-analysis
Source: Front Physiol. 2026 Apr 15;17:1797432. doi: 10.3389/fphys.2026.1797432 (PMC13126151; doi:10.3389/fphys.2026.1797432)
Supplement: Supplementary file 5 [file Table4.docx]

**Supplementary Table 4. Sensitivity analyses of ACSM adherence thresholds. (75%, 60%, 80% thresholds).**

| Outcome | Threshold | Subgroup | Studies (n) | SMD (95% CI) | *P*_within_ | *P*_between_ | *I*²(%) |
| --- | --- | --- | --- | --- | --- | --- | --- |
| Quality of Life | 75% (Primary) | High adherence | 9 | 0.75 [ 0.27, 1.23] | 0.002 | 0.03 | 88% |
|  |  | Low/Uncertain | 10 | 0.20 [ 0.05, 0.35] | 0.007 |  | 0% |
|  | 60% (Sensitivity) | High adherence | 15 | 0.54 [ 0.20, 0.87] | 0.002 | 0.07 | 82% |
|  |  | Low/Uncertain | 4 | 0.18 [ -0.00, 0.36] | 0.06 |  | 0% |
|  | 80% (Sensitivity) | High adherence | 5 | 0.88 [ -0.08, 1.84] | 0.07 | 0.24 | 92% |
|  |  | Low/Uncertain | 14 | 0.30 [ 0.18, 0.42] | <0.001 |  | 0% |
| Fatigue | 75% (Primary) | High adherence | 9 | -0.77 [ -1.17, -0.36] | <0.001 | 0.004 | 80% |
|  |  | Low/Uncertain | 5 | -0.11 [ -0.29, 0.07] | 0.23 |  | 0% |
|  | 60% (Sensitivity) | High adherence | 11 | -0.65[ -1.02,-0.28] | <0.001 | 0.01 | 79% |
|  |  | Low/Uncertain | 3 | -0.11[ -0.30,0.09] | 0.27 |  | 0% |
|  | 80% (Sensitivity) | High adherence | 4 | -1.04 [ -1.82, -0.26] | 0.009 | 0.07 | 78% |
|  |  | Low/Uncertain | 10 | -0.31 [ -0.49, -0.13] | <0.001 |  | 40% |
| Outcome | Threshold | Subgroup | Studies (n) | SMD (95% CI) | *P*_within_ | *P*_between_ | *I*²(%) |
| Anxiety | 75% (Primary) | High adherence | 9 | -0.74 [ -1.35, -0.14] | 0.02 | 0.37 | 92% |
|  |  | Low/Uncertain | 5 | -0.46 [ -0.65, -0.26] | <0.001 |  | 0% |
|  | 60% (Sensitivity) | High adherence | 13 | -0.65 [ -1.07, -0.22] | 0.003 | 0.53 | 88% |
|  |  | Low/Uncertain | 1 | -0.48 [ -0.77, -0.20] | 0.007 |  | N/A |
|  | 80% (Sensitivity) | High adherence | 6 | -0.76 [ -1.61, 0.09] | 0.08 | 0.61 | 93% |
|  |  | Low/Uncertain | 8 | -0.52 [ -0.83, -0.21] | 0.001 |  | 70% |
| Depression | 75% (Primary) | High adherence | 8 | -0.72 [ -1.22, -0.22] | 0.005 | 0.46 | 84% |
|  |  | Low/Uncertain | 5 | -0.52 [ -0.71, -0.32] | <0.001 |  | 0% |
|  | 60% (Sensitivity) | High adherence | 12 | -0.71 [ -1.05, -0.37] | <0.001 | 0.21 | 77% |
|  |  | Low/Uncertain | 1 | -0.43 [ -0.71, -0.15] | 0.003 |  | N/A |
|  | 80% (Sensitivity) | High adherence | 5 | -0.65 [ -1.46, 0.16] | 0.12 | 0.94 | 87% |
|  |  | Low/Uncertain | 8 | -0.68 [ -0.95, -0.42] | <0.001 |  | 57% |
| Outcome | Threshold | Subgroup | Studies (n) | SMD (95% CI) | *P*_within_ | *P*_between_ | *I*²(%) |
| Pain | 75% (Primary) | High adherence | 5 | -1.46 [ -2.57, -0.34] | 0.01 | 0.01 | 95% |
|  |  | Low/Uncertain | 4 | 0.02 [ -0.23, 0.28] | 0.87 |  | 0% |
|  | 60% (Sensitivity) | High adherence | 8 | -0.92 [ -1.70, -0.15] | 0.02 | 0.02 | 93% |
|  |  | Low/Uncertain | 1 | 0.06 [ -0.30, 0.42] | 0.75 |  | N/A |
|  | 80% (Sensitivity) | High adherence | 3 | -2.09 [ -3.94, -0.23] | 0.03 | 0.04 | 96% |
|  |  | Low/Uncertain | 6 | -0.17 [ -0.40, 0.06] | 0.14 |  | 18% |
| Sleep quality | 75% (Primary) | High adherence | 8 | -0.29 [ -0.57, -0.00] | 0.05 | 0.06 | 55% |
|  |  | Low/Uncertain | 4 | 0.18 [ -0.22, 0.58] | 0.38 |  | 61% |
|  | 60% (Sensitivity) | High adherence | 10 | -0.14 [ -0.45, 0.17] | 0.37 | 0.6 | 66% |
|  |  | Low/Uncertain | 2 | -0.03 [ -0.32, 0.27] | 0.86 |  | 22% |
|  | 80% (Sensitivity) | High adherence | 3 | -0.52 [ -1.03, -0.02] | 0.04 | 0.06 | 60% |
|  |  | Low/Uncertain | 9 | 0.00 [ -0.22, 0.22] | 1.00 |  | 39% |

Note: SMD, standardized mean difference; CI, confidence interval; *P*_within_ represents the statistical significance of the effect size within each subgroup; *P*_between_ represents the *P*-value for subgroup differences; *I^2^* represents the degree of heterogeneity within each subgroup. N/A (not applicable) indicates that heterogeneity could not be calculated because the subgroup contained only a single study.
