# Supplementary material for: Effects of ACSM guideline–based exercise on patients with lung cancer: a systematic review and meta-analysis
Source: Front Physiol. 2026 Apr 15;17:1797432. doi: 10.3389/fphys.2026.1797432 (PMC13126151; doi:10.3389/fphys.2026.1797432)
Supplement: Supplementary file 6 [file Table5.docx]

**Supplementary Table 5. Subgroup analyses by intervention duration and exercise type.**

| Outcome | Category | Subgroup | Studies (n) | SMD (95% CI) | *I*²(%) | *P_het_* | *P*_between_ |
| --- | --- | --- | --- | --- | --- | --- | --- |
| Quality of Life | Total | Overall effect | 19 | 0.44 [0.18, 0.70] | 80% | <0.001 |  |
|  | Duration | ≥12weeks | 9 | 0.34 [0.19, 0.49] | 7% | 0.37 | 0.68 |
|  |  | <12weeks | 10 | 0.46 [-0.07, 0.98] | 89% | <0.001 |  |
|  | Exercise Type | Combined | 10 | 0.31 [0.18, 0.45] | 0% | 0.60 | 0.39 |
|  |  | Single | 9 | 0.57 [0.01, 1.13] | 90% | <0.001 |  |
| Fatigue | Total | Overall effect | 14 | -0.50 [-0.81, -0.20] | 82% | <0.001 |  |
|  | Duration | ≥12weeks | 7 | -0.41 [-0.66, -0.16] | 50% | 0.06 | 0.65 |
|  |  | <12weeks | 7 | -0.56 [-1.18, 0.05] | 90% | <0.001 |  |
|  | Exercise Type | Combined | 8 | -0.21 [-0.36, -0.05] | 0% | 0.50 | 0.04 |
|  |  | Single | 6 | -0.80 [-1.35, -0.25] | 86% | <0.001 |  |
| Anxiety | Total | Overall effect | 14 | -0.63 [-1.00, -0.26] | 87% | <0.001 |  |
|  | Duration | ≥12weeks | 5 | -0.25 [-0.50, 0.00] | 41% | 0.15 | 0.05 |
|  |  | <12weeks | 9 | -0.87 [-1.43, -0.31] | 88% | <0.001 |  |
|  | Exercise Type | Combined | 7 | -0.36 [-0.77, 0.05] | 76% | <0.001 | 0.13 |
|  |  | Single | 7 | -0.92 [-1.51, -0.33] | 90% | <0.001 |  |
| Depression | Total | Overall effect | 13 | -0.67 [-0.97, -0.38] | 75% | <0.001 |  |
|  | Duration | ≥12weeks | 5 | -0.45 [-0.62, -0.28] | 0% | 0.70 | 0.09 |
|  |  | <12weeks | 8 | -0.94 [-1.48, -0.39] | 83% | <0.001 |  |
|  | Exercise Type | Combined | 7 | -0.58 [-0.96, -0.19] | 72% | 0.002 | 0.46 |
|  |  | Single | 6 | -0.82 [-1.35, -0.30] | 81% | <0.001 |  |
| Pain | Total | Overall effect | 9 | -0.81 [-1.49, -0.12] | 93% | <0.001 |  |
|  | Duration | ≥12weeks | 4 | -0.91 [-2.17, 0.36] | 97% | <0.001 | 0.80 |
|  |  | <12weeks | 5 | -0.71 [-1.44, 0.01] | 84% | <0.001 |  |
|  | Exercise Type | Combined | 4 | -0.25 [-0.57, 0.08] | 40% | 0.17 | 0.14 |
|  |  | Single | 5 | -1.24 [-2.52, 0.04] | 96% | <0.001 |  |
| Sleep quality | Total | Overall effect | 12 | -0.12 [-0.37, 0.12] | 62% | 0.002 |  |
|  | Duration | ≥12weeks | 8 | 0.06 [-0.18, 0.30] | 39% | 0.12 | 0.01 |
|  |  | <12weeks | 4 | -0.50 [-0.87, -0.13] | 52% | 0.10 |  |
|  | Exercise Type | Combined | 7 | -0.02 [-0.30, 0.25] | 52% | 0.05 | 0.35 |
|  |  | Single | 5 | -0.27 [-0.71, 0.17] | 69% | 0.01 |  |

Note: SMD, standardized mean difference; CI, confidence interval; *P*_het_ represents the *P*-value for heterogeneity within each subgroup; *P*_between_ represents the *P*-value for differences between subgroups; *I^2^* represents the percentage of heterogeneity within each subgroup.
