# Supplementary material for: Effects of ACSM guideline–based exercise on patients with lung cancer: a systematic review and meta-analysis
Source: Front Physiol. 2026 Apr 15;17:1797432. doi: 10.3389/fphys.2026.1797432 (PMC13126151; doi:10.3389/fphys.2026.1797432)
Supplement: Supplementary file 7 [file Table6.docx]

**Supplementary Table 6. Summary of findings and GRADE certainty of evidence.**

| Outcome (measurement: post-intervention values) | No. of RCTs (Total participants, Intervention/Control) | Pooled effect estimate (95% CI) | Certainty of evidence (GRADE) | Key reasons for downgrading |
| --- | --- | --- | --- | --- |
| Primary outcomes |  |  |  |  |
| Quality of Life (EORTC QLQ-C30, SF-36, FACT-L) | 19 RCTs (N=1444; 728/716) | SMD 0.44 (0.18 to 0.70), *p* < 0.001 | Low | ① Serious risk of bias (all studies open-label, patients unblinded; high risk of performance/detection bias); ② Serious inconsistency (*I²*=80%, high heterogeneity) |
| Secondary outcomes: symptom-related and psychological outcomes |  |  |  |  |
| Fatigue (FACIT-F, BFI, PFS, EORTC fatigue subscale) | 14 RCTs (N=1144; 568/576) | SMD −0.50 (−0.81 to −0.20), *p* = 0.001 | Low | ① Serious risk of bias (all studies open-label; subjective outcome); ② Serious inconsistency (*I²*=82%, high heterogeneity) |
| Anxiety (HADS-A, SAS, GAD-7) | 14 RCTs (N=1105; 552/553) | SMD −0.63 (−1.00 to −0.26), *p* < 0.001 | Low | ① Serious risk of bias (unblinded patients, subjective outcome); ② Serious inconsistency (*I²*=87%, high heterogeneity) |
| Depression (HADS-D, SDS, PHQ-9) | 13 RCTs (N=949; 474/475) | SMD −0.67 (−0.97 to −0.38), *p* < 0.001 | Low | ① Serious risk of bias (unblinded patients, subjective outcome); ② Serious inconsistency (*I²*=75%, high heterogeneity) |
| Pain (EORTC pain subscale, VAS, NRS) | 9 RCTs (N=613; 305/308) | SMD −0.81 (−1.49 to −0.12), *p* = 0.02 | Very low | ① Serious risk of bias (unblinded patients, subjective outcome); ② Serious inconsistency (*I²*=93%, extreme heterogeneity); ③ Serious imprecision (95% CI wide, total sample size < OIS) |
| Sleep Quality (PSQI, EORTC insomnia subscale) | 12 RCTs (N=788; 392/396) | SMD −0.12 (−0.37 to 0.12), *p* = 0.32 | Very low | ① Serious risk of bias (unblinded patients, subjective outcome); ② Serious inconsistency (*I²*=62%, moderate heterogeneity); ③ Serious imprecision (95% CI crosses null, no statistically significant effect) |

Note: SMD = standardized mean difference; CI = confidence interval; RCT = randomized controlled trial; *I²* = heterogeneity statistic.
