# Supplementary material for: Effects of ACSM guideline–based exercise on patients with lung cancer: a systematic review and meta-analysis
Source: Front Physiol. 2026 Apr 15;17:1797432. doi: 10.3389/fphys.2026.1797432 (PMC13126151; doi:10.3389/fphys.2026.1797432)
Supplement: Supplementary file 8 [file DataSheet1.docx]

**Supplementary Material: List of Forest Plots for Sensitivity Analyses**

Supplementary Figures 1-1 to 1-12.

Supplementary Figure 1-1 Forest plot: Quality of Life (60% threshold)

Supplementary Figure 1-2 Forest plot: Fatigue (60% threshold)

Supplementary Figure 1-3 Forest plot: Anxiety (60% threshold)

Supplementary Figure 1-4 Forest plot: Depression (60% threshold)

Supplementary Figure 1-5 Forest plot: Pain (60% threshold)

Supplementary Figure 1-6 Forest plot: Sleep quality (60% threshold)

Supplementary Figure 1-7 Forest plot: Quality of Life (80% threshold)

Supplementary Figure 1-8 Forest plot: Fatigue (80% threshold)

Supplementary Figure 1-9 Forest plot: Anxiety (80% threshold)

Supplementary Figure 1-10 Forest plot: Depression (80% threshold)

Supplementary Figure 1-11 Forest plot: Pain (80% threshold)

Supplementary Figure 1-12 Forest plot: Sleep quality (80% threshold)


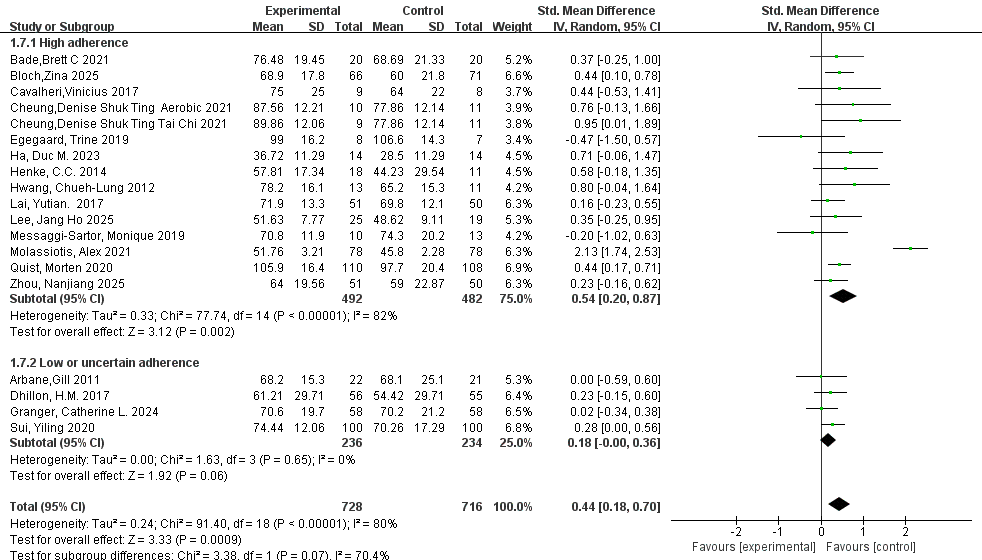


Supplementary Figure 1-1. Sensitivity analysis of Quality of Life (Threshold = 60%).


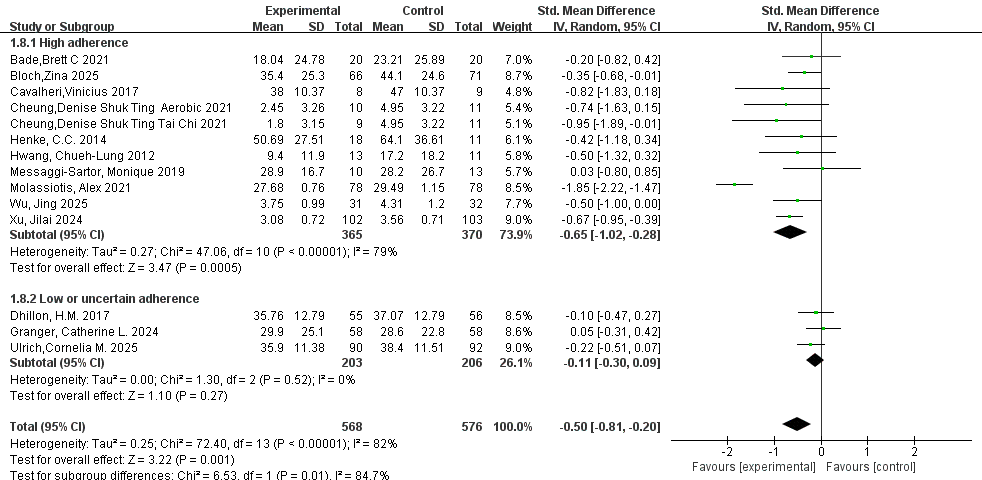


Supplementary Figure 1-2. Sensitivity analysis of Fatigue (Threshold = 60%).


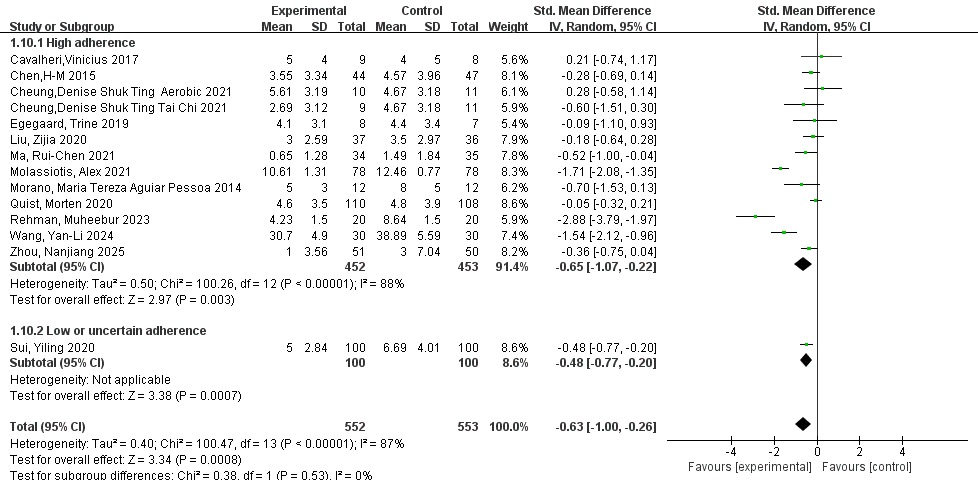


Supplementary Figure 1-3. Sensitivity analysis of Anxiety (Threshold = 60%).


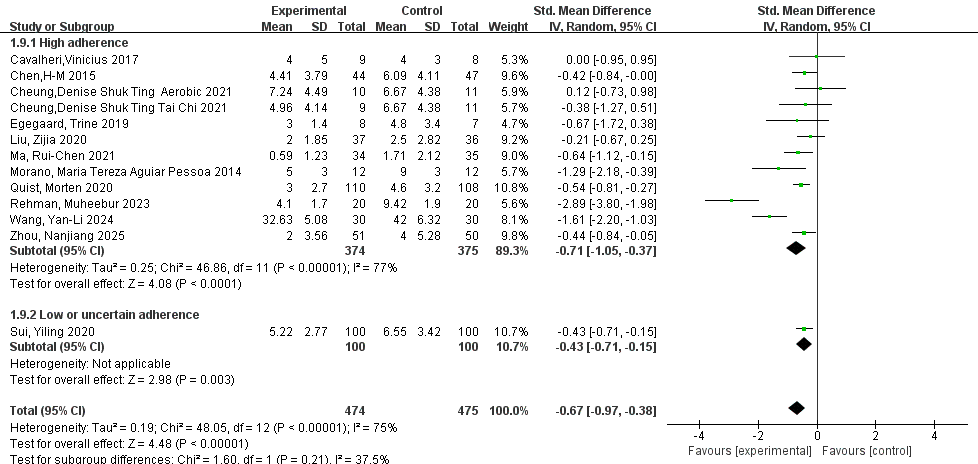


Supplementary Figure 1-4. Sensitivity analysis of Depression (Threshold = 60%).


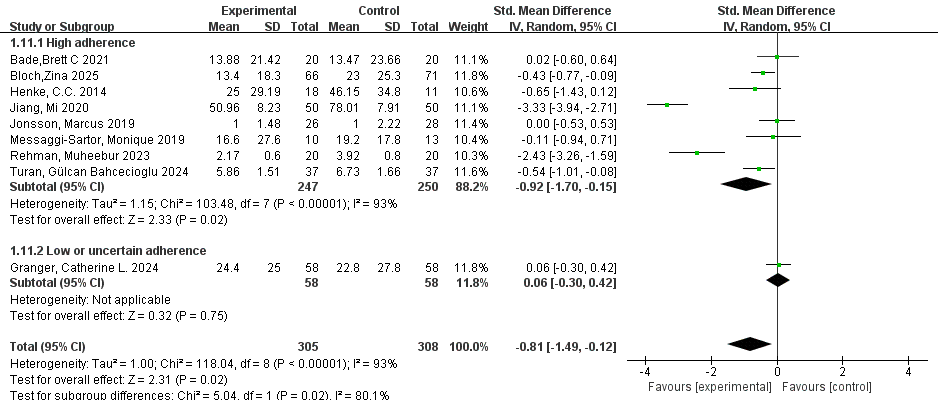


Supplementary Figure 1-5. Sensitivity analysis of Pain (Threshold = 60%).


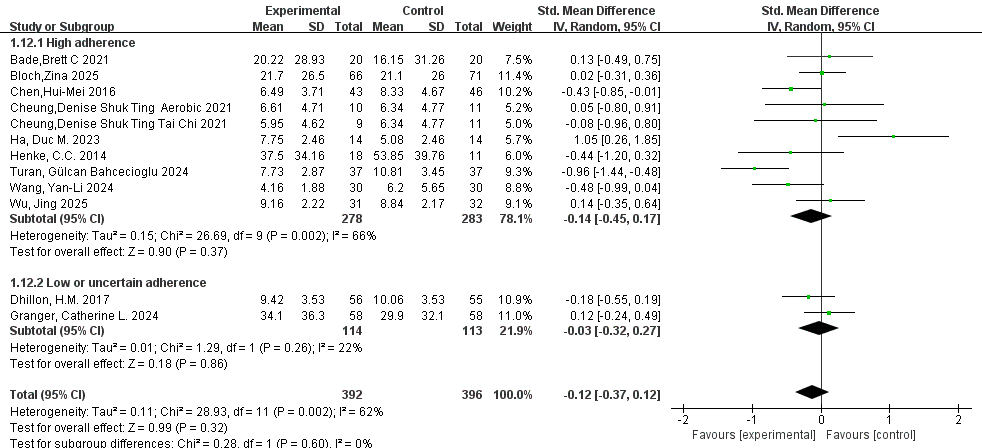


Supplementary Figure 1-6. Sensitivity analysis of Sleep quality (Threshold = 60%).


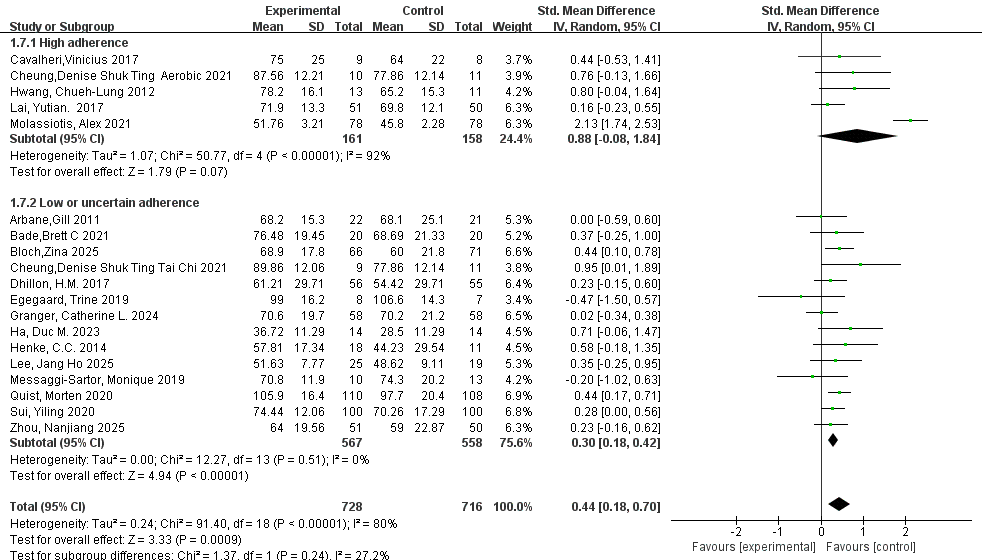


Supplementary Figure 1-7. Sensitivity analysis of Quality of Life (Threshold = 80%).


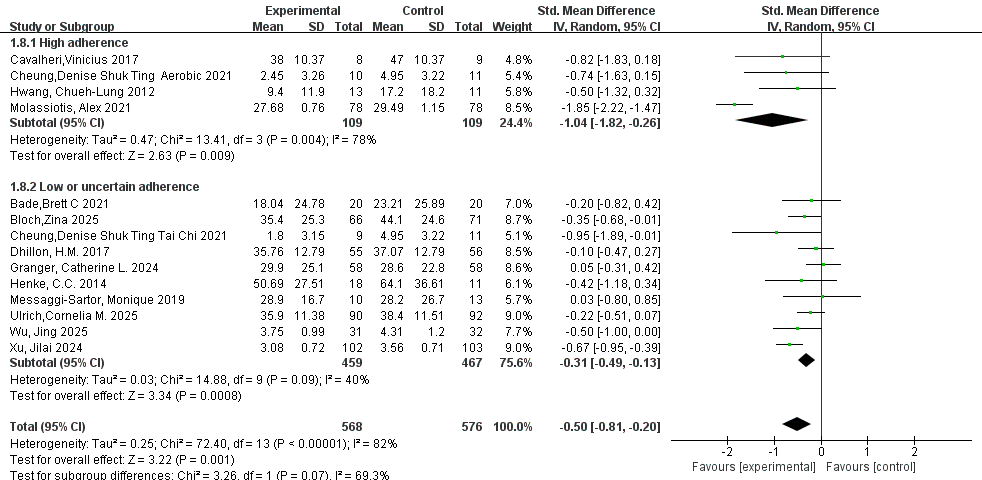


Supplementary Figure 1-8. Sensitivity analysis of Fatigue (Threshold = 80%).


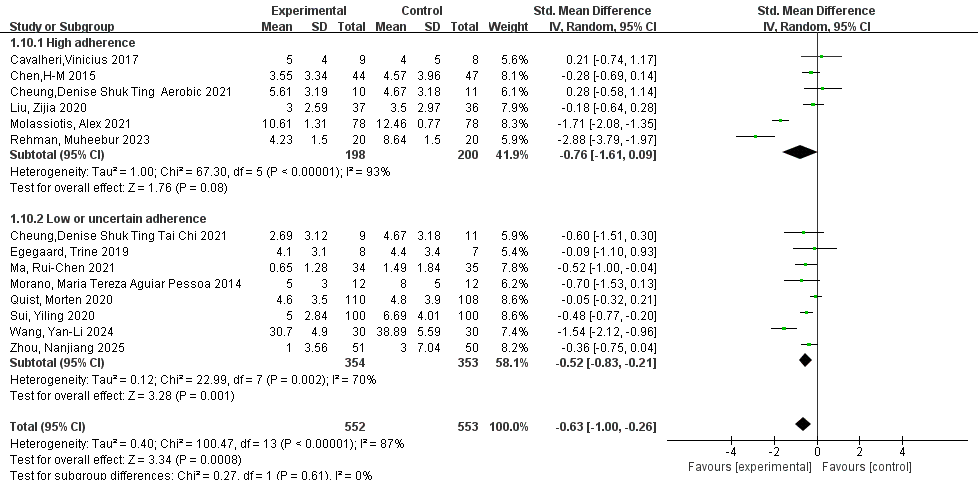


Supplementary Figure 1-9. Sensitivity analysis of Anxiety (Threshold = 80%).


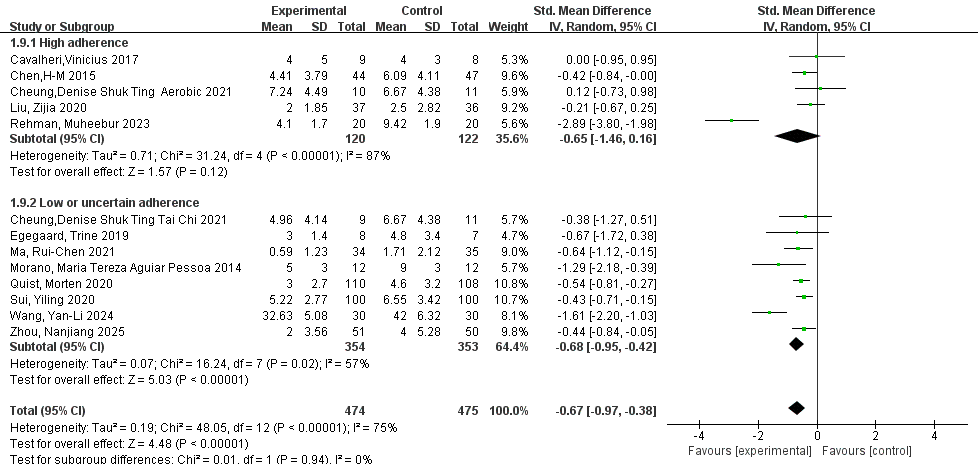


Supplementary Figure 1-10. Sensitivity analysis of Depression (Threshold = 80%).


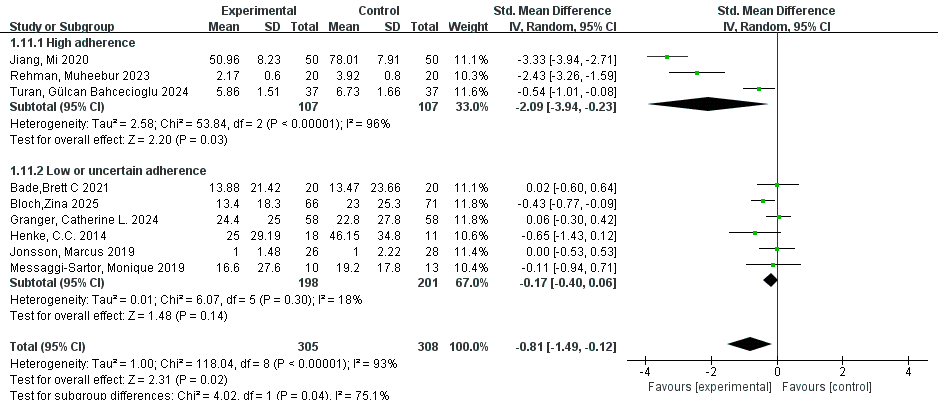


Supplementary Figure 1-11. Sensitivity analysis of Pain (Threshold = 80%).


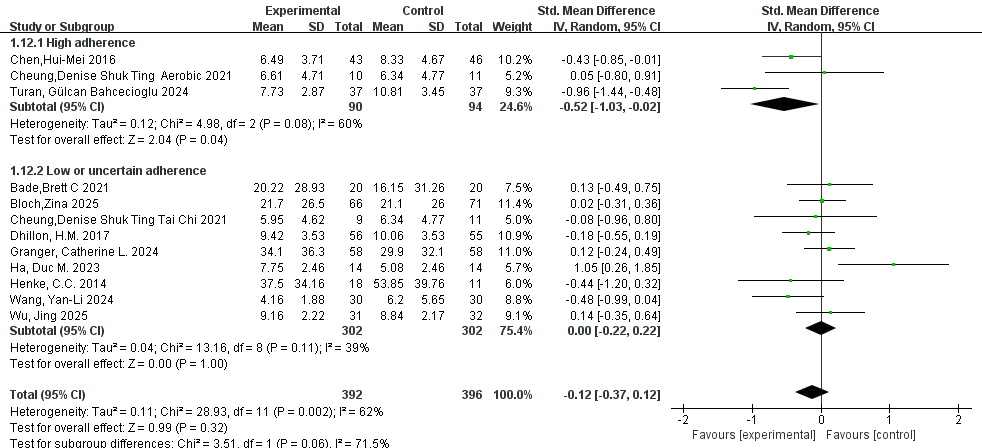


Supplementary Figure 1-12. Sensitivity analysis of Sleep quality (Threshold = 80%).
