# Supplementary material for: Effects of ACSM guideline–based exercise on patients with lung cancer: a systematic review and meta-analysis
Source: Front Physiol. 2026 Apr 15;17:1797432. doi: 10.3389/fphys.2026.1797432 (PMC13126151; doi:10.3389/fphys.2026.1797432)
Supplement: Supplementary file 9 [file DataSheet2.docx]

**Supplementary Material: List of Subgroup Analysis Forest Plots**

Supplementary Figures 2-1 to 2-12

Part A: Subgroup Analysis by Intervention Duration (≥12 weeks vs. <12 weeks)

Supplementary Figure 2-1 Forest plot: Quality of Life (Intervention Duration)

Supplementary Figure 2-2 Forest plot: Fatigue (Intervention Duration)

Supplementary Figure 2-3 Forest plot: Anxiety (Intervention Duration)

Supplementary Figure 2-4 Forest plot: Depression (Intervention Duration)

Supplementary Figure 2-5 Forest plot: Pain (Intervention Duration)

Supplementary Figure 2-6 Forest plot: Sleep quality (Intervention Duration)

Part B: Subgroup Analysis by Exercise Type (Combined vs. Individual Exercise)

Supplementary Figure 2-7 Forest plot: Quality of Life (Exercise Type)

Supplementary Figure 2-8 Forest plot: Fatigue (Exercise Type)

Supplementary Figure 2-9 Forest plot: Anxiety (Exercise Type)

Supplementary Figure 2-10 Forest plot: Depression (Exercise Type)

Supplementary Figure 2-11 Forest plot: Pain (Exercise Type)

Supplementary Figure 2-12 Forest plot: Sleep quality (Exercise Type)


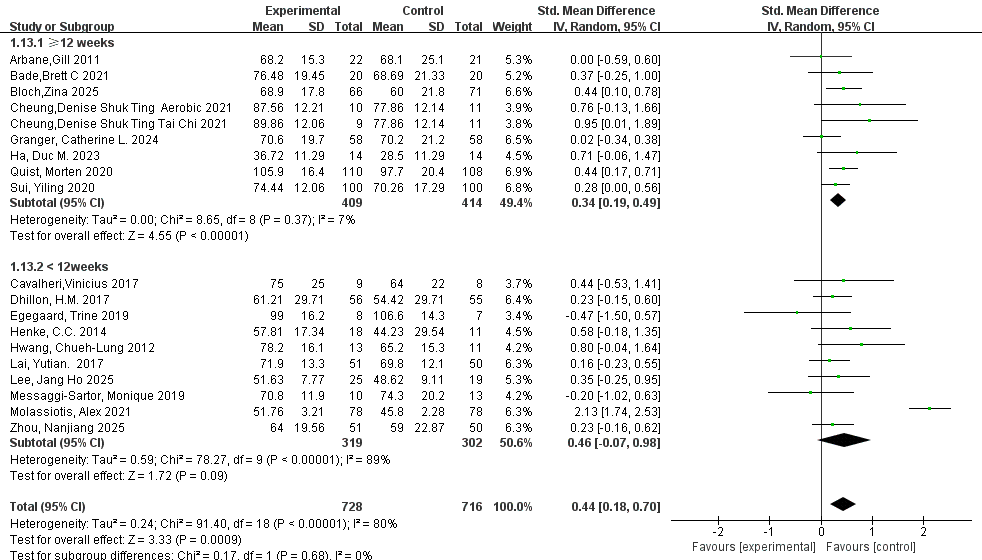


Supplementary Figure 2-1 Forest plot: Quality of Life (Intervention Duration)


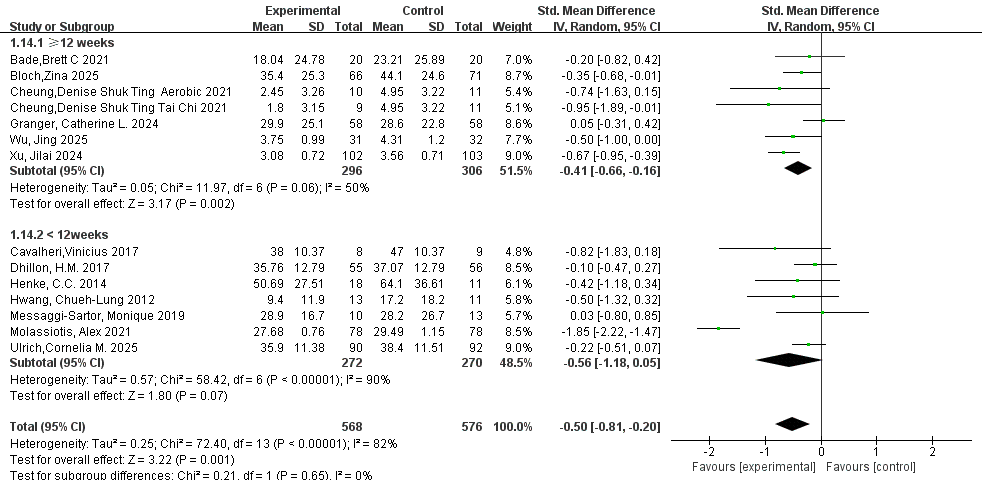


Supplementary Figure 2-2 Forest plot: Fatigue (Intervention Duration)


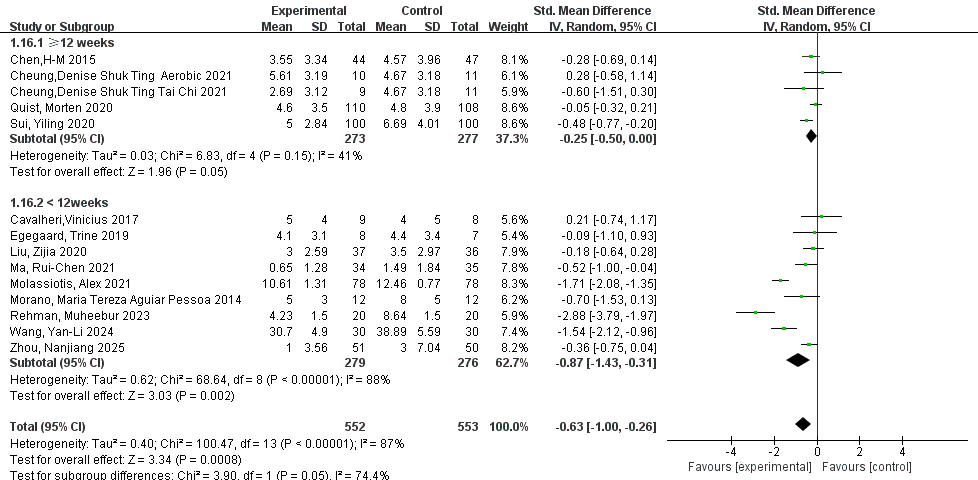


Supplementary Figure 2-3 Forest plot: Anxiety (Intervention Duration)


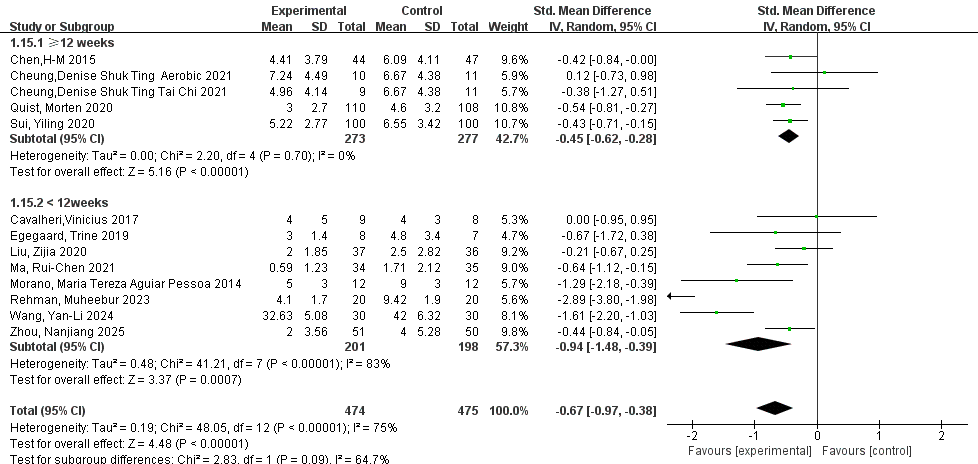


Supplementary Figure 2-4 Forest plot: Depression (Intervention Duration)


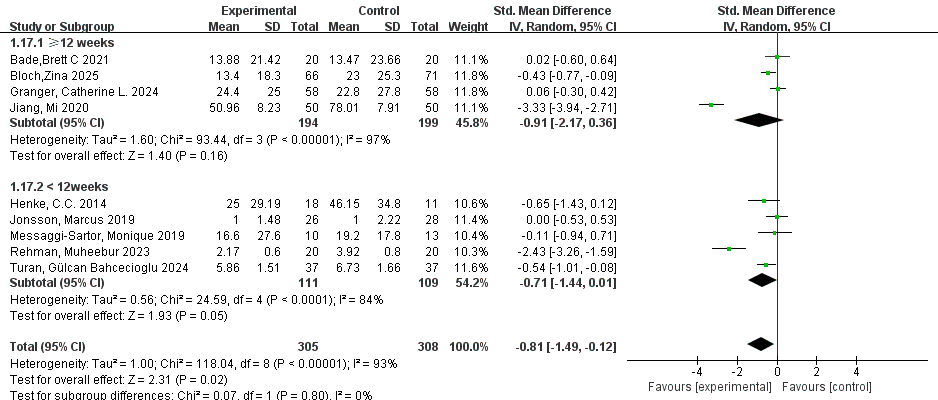


Supplementary Figure 2-5 Forest plot: Pain (Intervention Duration)


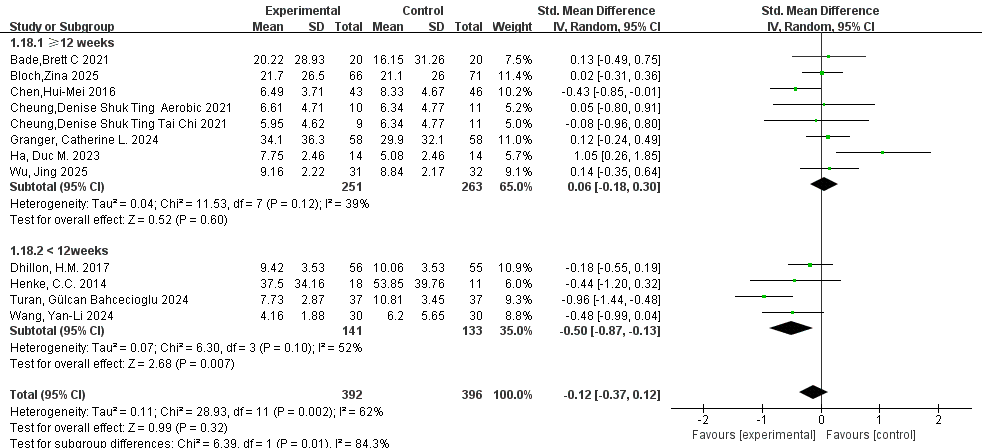


Supplementary Figure 2-6 Forest plot: Sleep quality (Intervention Duration)


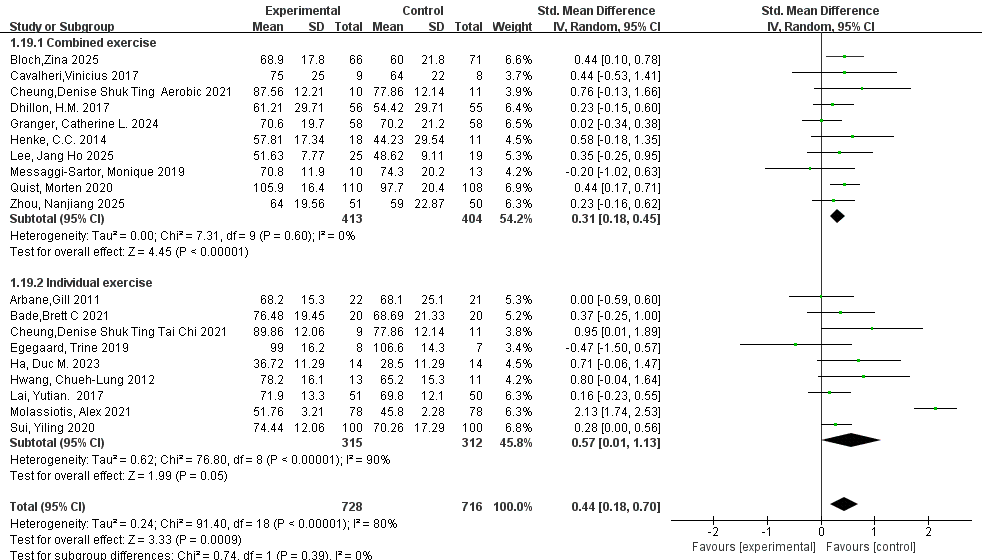


Supplementary Figure 2-7 Forest plot: Quality of Life (Exercise Type)


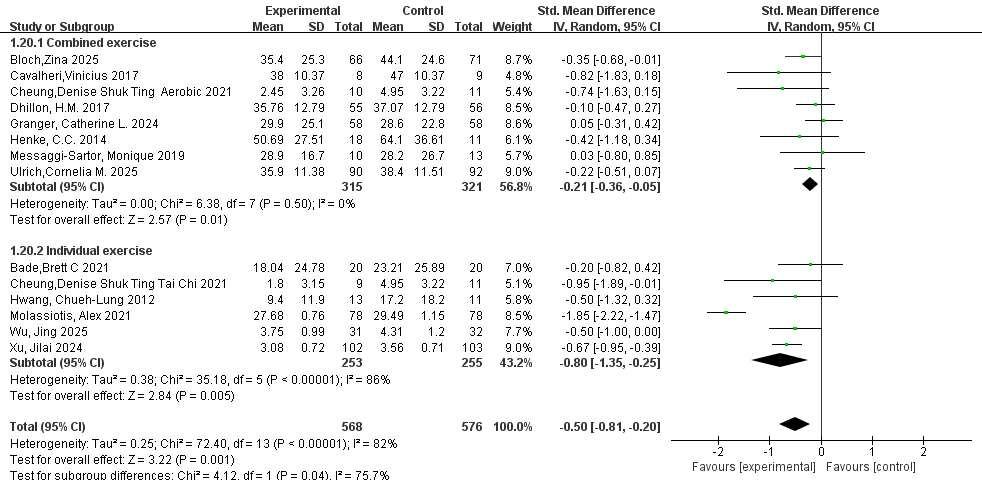


Supplementary Figure 2-8 Forest plot: Fatigue (Exercise Type)


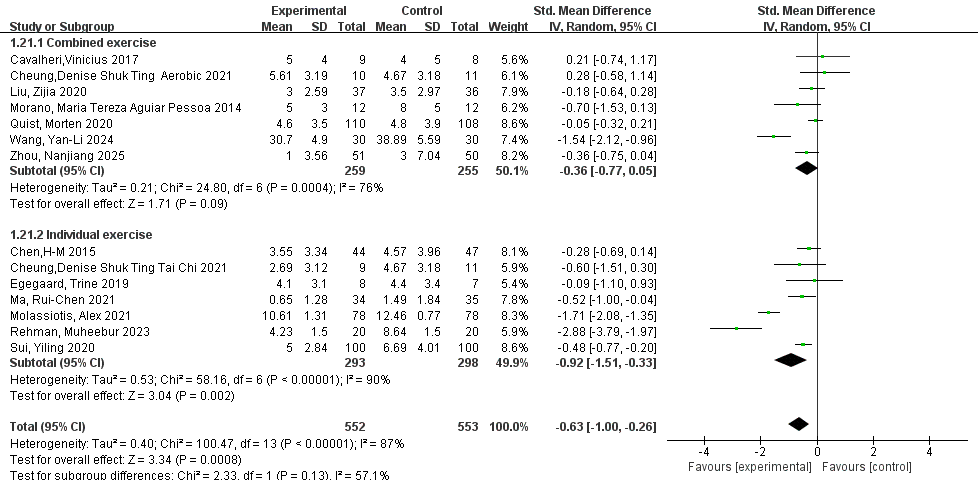


Supplementary Figure 2-9 Forest plot: Anxiety (Exercise Type)


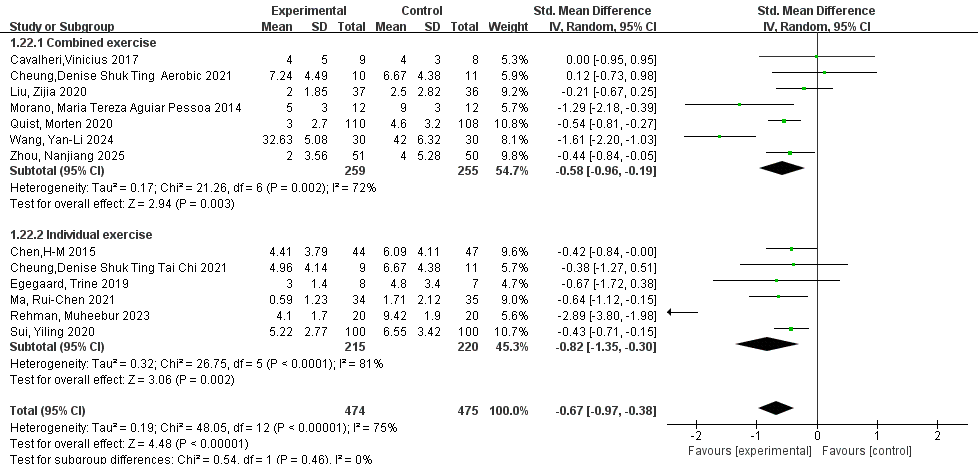


Supplementary Figure 2-10 Forest plot: Depression (Exercise Type)


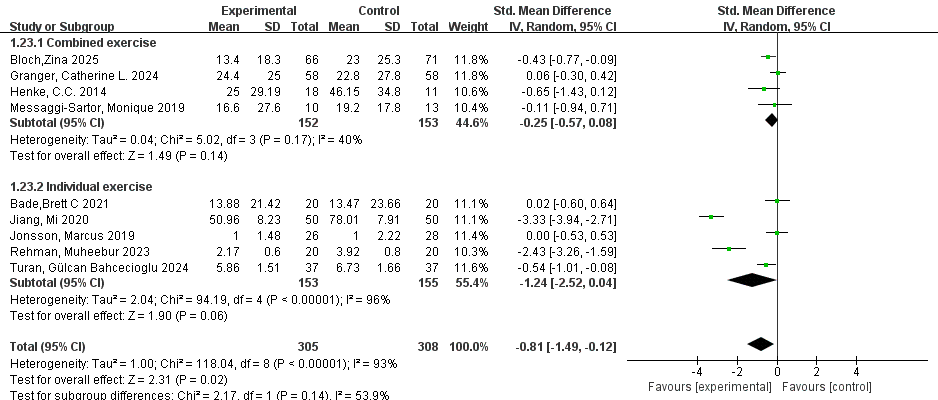


Supplementary Figure 2-11 Forest plot: Pain (Exercise Type)


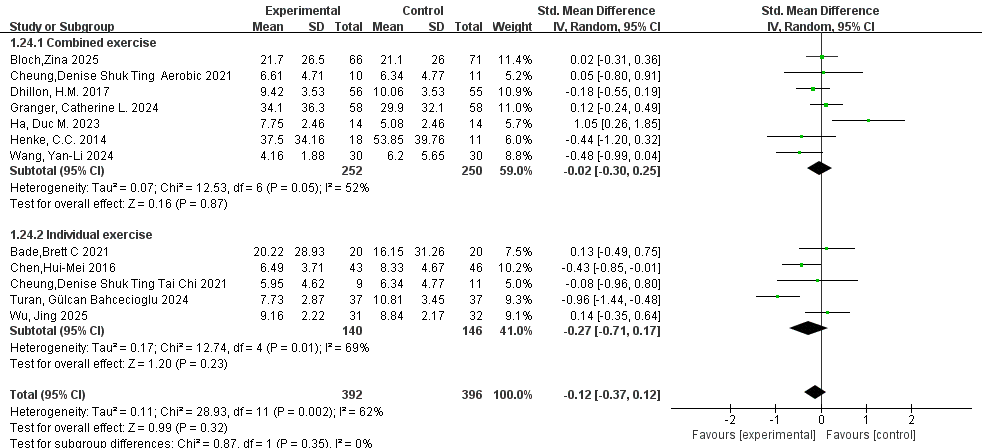


Supplementary Figure 2-12 Forest plot: Sleep quality (Exercise Type)
